# Genome-wide reprogramming of sRNA and lncRNA in the epigenetic regulation following interspecific hybridization in the *Brassica* species

**DOI:** 10.1186/s43897-025-00194-8

**Published:** 2026-02-11

**Authors:** Chengtao Quan, Ling Wang, Hangpan Wei, Yilin Ma, Yunqin Wang, Guoting Cheng, Chaozhi Ma, Cheng Dai

**Affiliations:** 1 National Key Laboratory of Crop Genetic Improvement, Huazhong Agricultural University, Wuhan, 430070 China; 2 Biobreeding Institute, Xianghu Laboratory, Hangzhou, 311231 China; 3 College of Informatics, Huazhong Agricultural University, Wuhan, 430070 China; 4 Hubei Hongshan Laboratory, Wuhan, 430070 China

**Keywords:** Brassicaceae, Interspecific hybridization, Non-coding RNAs, DNA methylation, Chromatin accessibility

## Abstract

Interspecific hybridization has a significant impact on the inheritance of non-coding RNAs (ncRNAs), resulting in alterations in gene expression and phenotypic variation. However, the mechanisms behind interspecific hybridization-mediated ncRNA regulation and its interaction with epigenetic pathways are not well understood. We examined ncRNA profiles in two F_1_ hybrids of *Brassica napus* and *Brassica rapa* (Hybrid-sh and Hybrid-yh). Regulatory differences in small RNAs (sRNAs) between hybrids were mainly driven by maternal inheritance, with Hybrid-sh exhibiting more substantial maternal influence and Hybrid-yh showing transgressive regulation, while 24-nt siRNAs were directly involved in methylation control. We identified 47,855 lncRNA transcripts exhibiting more pronounced non-additive expression patterns during interspecific hybridization than protein-coding mRNAs. Intergenic lncRNAs maintained higher methylation levels across CG/CHG/CHH contexts but had less chromatin accessibility than antisense lncRNAs. Notably, *lncRNA0410*, a conserved antisense transcript from *CP12-1*'s 5′-region, contained a chromatin-accessible site in Hybrid-yh with limited conservation beyond crucifers. It enhanced flavonol biosynthesis by activating *BnaA03.MYB12*, improving drought tolerance. Intriguingly, dosage-sensitive lncRNAs exhibited unique epigenetic signatures, including siRNA enrichment, methylation, and chromatin accessibility features that influence transcriptional responses. This research sheds light on the roles of ncRNA in hybrid vigor and genome stability, revealing key epigenetic regulatory mechanisms in plant hybridization.

**Supplementary Information:**

The online version contains supplementary material available at 10.1186/s43897-025-00194-8.

## Core

Following interspecies hybridization, the expression levels of non-coding RNAs change rapidly. The differential expression of 24-nt siRNA clusters in F_1_ hybrids directly influences the changes in DNA methylation levels. Long non-coding RNAs (lncRNAs) mediated by chromatin-accessible regions influence gene expression in a *cis*-regulatory manner. ACR-*lncRNA0410*, identified in the A subgenome, positively regulates *BnaA03.MYB12* expression promotes the accumulation of flavonols and enhances plant drought resistance.

## Gene and accession numbers

All high-throughput sequencing data for RNA-seq, small RNA-seq, ATAC-seq, and WGBS of *Brassica* allotriploid hybrids and parents in this study are available in the Short Read Archive (SRA) under NCBI BioProject accession number PRJNA1154317. The raw data can also be queried at https://gigadb.org/dataset/102668.

## Introduction

Interspecific hybridization occurs when two distinct species mate, resulting in offspring that carry a mix of genetic materials from both parents. This exchange of genes can introduce new traits and alleles, which may offer selective benefits like improved fitness, resilience to environmental challenges, or the capability to occupy different ecological niches (Dong et al. 2018; Huang et al. 2016; Zhang et al. 2021). While prior research has predominantly concentrated on genes, there has been a growing recognition of the significance of non-coding RNA (ncRNA) in interspecific hybridization. Numerous recent studies have substantiated that non-coding RNA plays a crucial role in these processes (Borges et al. 2021; Palazzo et al. 2020; Rogers et al. 2013).

Non-coding RNAs, which encompass long non-coding RNAs (lncRNAs) and small RNAs (sRNAs), fulfill various roles in cellular processes (Roulé et al. 2022; Wang et al. 2022). LncRNAs, typically over 200 nucleotides, are essential for chromatin remodeling and regulating transcription and post-transcription processes. They significantly contribute to plant development, responses to stress, and epigenetic modifications (Cheng et al. 2023; Du et al. 2025). LncRNAs can influence nearby genes in a *cis*-regulatory manner or have a broader trans-regulatory impact (Wang et al. 2018). They also modulate gene expression by recruiting histone-modifying complexes to targeted genomic locations, leading to either gene activation or repression (Werner et al. 2017; Imaduwage and Hewadikaram 2024). The hybridization process to the genome initiates the reprogramming of the lncRNA transcriptome in F_1_ hybrids. In cotton, after hybridization and whole-genome duplication (WGD), a long intergenic non-coding RNA, *DAN1*, which is involved in regulating drought stress, arises from the A diploid genome of the cotton ancestor (Tao et al. 2021). This implies that interspecific hybridization and WGD contribute to genomic alterations that facilitate the emergence of new functions in non-coding genes during natural evolution. Furthermore, these activated lncRNAs primarily originate from demethylated transposable element (TE) regions, and the changes in DNA methylation that occur with interspecific hybridization are closely associated with variations in lncRNA expression in cotton post-hybridization (Zhao et al. 2018). This underscores the importance of lncRNAs in sustaining genetic variation stability and enhancing genetic diversity.

In plants, endogenous sRNAs range from 21 to 25 nucleotides (nt) (Axtell 2013). The 21-nt sRNAs predominantly comprise microRNAs (miRNAs) that regulate mRNA expression, while 24-nt sRNAs are associated with RNA-mediated DNA methylation (Satyaki et al. 2019; Vaucheret et al. 2024). Both miRNAs and small interfering RNAs (siRNAs) play essential roles in the RNA interference (RNAi) pathway, where sRNA molecules direct Argonaute proteins to complementary RNA sequences, leading to either degradation or repression of the target RNA's translation (He et al. 2021; Weiberg et al. 2013). Notably, a greater proportion of non-additively expressed miRNAs has been noted in interspecific hybrids or allopolyploids of *Arabidopsis*, wheat, and maize (Crisp et al. 2020; Ha et al. 2009; Li et al. 2014a). This observation results in variations in the expression of key target genes, significantly influencing the growth, vitality, and adaptability of F_1_ hybrids (Li et al. 2021). Furthermore, the abundance of 21-nt, 22-nt, and 24-nt siRNAs in maize hybrids shows minimal variation between inbred lines and hybrids, with hybridization primarily impacting sRNA expression at specific loci rather than on a broader scale (Crisp et al. 2020). siRNAs have been associated with alterations in DNA methylation and gene expression in hybrids, likely as a protective mechanism against genomic instability and regulating gene expression via DNA methylation modulation (Borges et al. 2021; Erdmann et al. 2020). These small RNAs may serve a regulatory function in balancing the parental genomes and controlling gene expression in triploid Arabidopsis plants, which arise from a diploid maternal parent and a tetraploid paternal parent, ultimately leading to larger seed size (Lu et al. 2012).

The Brassicaceae family comprises essential flowering plants that yield vegetable oils for human and industrial purposes (Chalhoub et al. 2014; Zhou et al. 2024). Despite this significance, there remains limited knowledge regarding the role of non-coding RNAs in influencing genetic recombination in plants, particularly within the Brassicaceae, during interspecific hybridization. This study examined the genetic and genomic impact of long non-coding RNAs (lncRNAs) and small RNAs (sRNAs) in hybrids of *Brassica napus* and *Brassica rapa*. We specifically investigated the association of these non-coding RNAs with DNA methylation and chromatin accessibility. As research expands on non-coding RNAs and epigenetic regulation, we expect these non-coding RNAs to shed light on hybrid vigor, genome compatibility, and the inheritance of traits.

## Results

### Rapid changes in sRNA expression levels after interspecific hybridization.

**Fig. 1:**
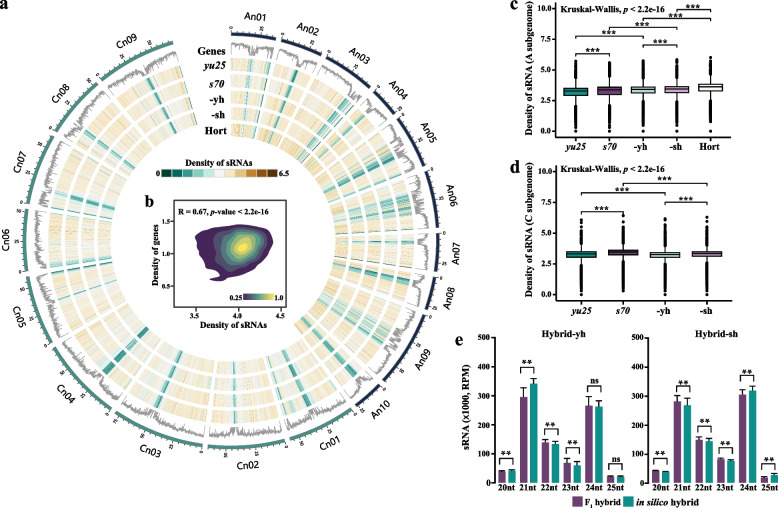
Small RNAs distribution in F_1_ hybrids and parental lines. **a** Browser Circos plots showed the genome density distribution of genes and sRNAs at a resolution of 100 kb, with the top track indicating gene density. *yu25*, *s70*, -yh, -sh, and Hort represent the distribution of sRNAs, respectively. The suffixes -sh and -yh refer to Hybrid-sh and Hybrid-yh, respectively. **b** Pairwise comparison of genes and sRNAs density. R is the Pearson's correlation coefficient. The color scale measures the density of points. **c-d** Density of sRNAs in the A (**c**) and C (**d**) subgenomes in *yu25*, *s70*, -yh, -sh, and Hort. The suffixes -sh and -yh refer to Hybrid-sh and Hybrid-yh, respectively. The Kruskal–Wallis test was employed to determine significant differences. **e** Histograms showed the distribution of sRNAs in F_1_ hybrids and in silico hybrids. ANOVA performed statistical tests. Different letters indicate significant differences (*p* < 0.01). Error bars indicated the mean ± SD of three biological replicates

Hybridization has been shown to activate transposable elements (TEs), disrupting small RNA (sRNA) stability and altering gene expression (Lopez-Gomollon et al. 2022). To investigate sRNA resetting after interspecific hybridization between *B. napus* and *B. rapa*, two allotriploid *Brassica* hybrids were generated by crossing inbred *B. napus* lines (maternal) (*s70* [genome: A_s_A_s_C_s_C_s_] and *yu25* [A_y_A_y_C_y_C_y_]) with a *B. rapa* line (paternal) (Hort [A_h_A_h_]). The resulting hybrids, Hybrid-sh (*s70* × Hort, A_h_A_s_C_s_) and Hybrid-yh (*yu25* × Hort, A_h_A_y_C_y_), and their parental lines (Fig. S1), were then performed small RNA (sRNA) sequencing and yielded an average of 20 million high-quality reads per replicate, with 89.4% successfully mapped to the reference genome (Table S1). These sRNAs were predominantly enriched in euchromatic regions, and their distribution positively correlated with gene density (R = 0.67; Figs. 1a, b). In the A subgenome, the sRNA density across the genome in F_1_ hybrids was higher than in the maternal but lower than in the paternal (Fig. 1c). In contrast, the density of hybrids' sRNA in the C subgenome was lower than that of the maternal (Fig. 1d). Moreover, Hybrid-sh showed significantly higher sRNA density compared to Hybrid-yh in both the A and C subgenomes (Kruskal–Wallis, *p* < 2.2e-16) (Figs. 1c, d). These results suggest that the dynamic expression of sRNAs mediated by genomic recombination could modulate transcriptional activation or suppression.

To assess hybridization-induced changes in sRNA profiles, we generated in silico hybrids (in silico-sh and in silico-yh) by computationally merging maternal and paternal sRNA-seq datasets at a 1:1 ratio. We found that both in silico hybrids had lower expression levels than F_1_ hybrids for the 22-nt and 23-nt sRNA (Fig. 1e). However, more diverse patterns were observed in 20-, 21-, 24-, and 25-nt sRNAs between in silico and F_1_ hybrids. For example, Hybrid-yh exhibited reduced levels of 21-nt sRNA expression compared to in silico-yh, whereas Hybrid-sh showed significantly elevated levels relative to in silico-sh; conversely, the expression of 24-nt sRNA in Hybrid-yh was similar to that in silico-yh, while Hybrid-sh displayed lower expression than in silico-sh (Fig. 1e). These findings demonstrate that interspecific hybridization exerts genotype-dependent effects on the length-specific regulation of sRNA.

### siRNA loci with varying lengths in F_1_ hybrids and their parental lines

We then analyzed siRNA loci density in F_1_ hybrids and their parental lines to investigate variations in siRNA cluster abundance at specific genetic loci. The total number of identified siRNA clusters was 47,449 (*yu25*), 66,063 (*s70*), 51,119 (Hybrid-yh), 58,009 (Hybrid-sh), and 44,190 (Hort) (Tables S2–S6). In all categories, 21-nt and 24-nt siRNA clusters showed greater enrichment than others, with 24-nt siRNA clusters being more prevalent than 21-nt clusters (Fig. S2a). Therefore, we examined the 21-nt and 24-nt siRNA clusters in the F_1_ hybrids and their parent lines. The results showed that Hybrid-yh exhibited 24-nt siRNA clusters comparable to its paternal parent (Hort) but higher than its maternal parent (*yu25*), whereas Hybrid-sh displayed a divergent trend (Fig. S2a), suggesting genetic background complexity influences siRNA cluster formation. siRNA clusters were then categorized by genomic location into genic, proximal (< 2 kb from genes), and distal (> 2 kb from genes). Both 21-nt and 24-nt clusters were predominantly proximally localized (Fig. S2b).

The 24-nt siRNAs guide DNA methylases to TE regions, leading to methylation that reduces transcription and stabilizes the genome (Ziegler et al. 2023). Approximately 60% of 24-nt siRNA clusters originated from transposable elements (TEs), with DNA elements representing the largest subset (15.9%) (Fig. S2b). The F_1_ hybrids showed distinct patterns of 24-nt siRNA cluster density relative to parental lines, especially near gene bodies, following a trend of maternal > hybrid > paternal (Kruskal–Wallis, *p* < 2.2e-16); in contrast, 24-nt siRNA clusters associated with transposons revealed no significant differences due to hybridization (Fig. S2c), indicating that the accumulation dynamics of 24-nt siRNA in hybrids preferentially influence genic regions rather than TE regions.

### Distribution of various lengths of miRNAs and phasiRNAs throughout the genome

Plant small RNAs (sRNAs) are short regulatory molecules spanning 20—25 nucleotides (nt) in length (Axtell 2013). MicroRNAs (miRNAs), for example, are typically 21-nt long and suppress gene expression by binding complementary sequences in the 3′ untranslated region (UTR) of target mRNAs (Yu et al. 2017). In contrast, siRNAs mediate DNA methylation and histone modifications, which suppress gene expression at the transcriptional level and promote genomic stability associated with transposons and repetitive sequences (Li et al. 2020). We identified 343 miRNAs (derived from single-stranded precursors with hairpin structures) and 5,678 phasiRNAs (derived from Dicer/Dicer-like processing of double-stranded RNAs) across all lines (Tables S7, S8).

Among miRNAs, 21-nt miRNAs were the most prevalent, comprising 72.6% of the total (Table S7). Next were 22-nt miRNAs at 15.1%, followed by 20-nt miRNAs at 11.3%; in contrast, 24-nt miRNAs were the least common, representing merely 1.1% of the overall group (Table S7). We also identified 1,736 21-nt phasiRNAs and 3,942 24-nt phasiRNAs across all samples (Table S8). For 21- and 24-nt phasiRNAs, 59.2% and 44.9% were enriched in promoter regions, respectively (Fig. S3a). However, in distal regions, 24-nt phasiRNAs outnumbered 21-nt phasiRNAs (51.4% vs. 33.4%). A significant difference was noted in genic elements: 7.2% of 21-nt phasiRNAs were located in exons, compared to only one 24-nt phasiRNA found in these regions (Fig. S3a). Conversely, 3.7% of 24-nt phasiRNAs were identified in intronic areas, while only three 21-nt phasiRNAs were detected in introns (Fig. S3a).

Furthermore, compared to the parental lines, we identified 95 activated phasiRNAs in Hybrid-sh and 266 in Hybrid-yh, along with 156 and 235 silenced phasiRNAs, respectively (Fig. S3b). This was determined by the criteria that activated phasiRNAs had reads > 1 in F_1_ hybrids and reads = 0 in their parental lines, while silenced phasiRNAs had reads = 0 in F_1_ hybrids and reads > 1 in their parental lines. These results indicate that Hybrid-yh is more prone to genomic rearrangements arising from interspecific hybridization. Further analysis showed that around 55% of activated phasiRNAs were in promoter regions, and 43% were in the distal areas (Fig. S3b). Conversely, the distribution of silenced phasiRNAs in promoter and distal regions displayed no significant difference (181 vs. 186) (Fig. S3b).

### miRNAs and phasiRNAs that are expressed differently in F_1_ hybrids

The altered miRNA and siRNA expression in hybrids regulates target genes, ensures genomic stability, and impacts plant growth (Li et al. 2021; Shi et al. 2022). To assess hybridization effects on miRNA/phasiRNA dynamics, we analyzed miRNA and phasiRNA expression changes in F_1_ hybrids and their parents. Compared to the parentals, the F_1_ hybrids exhibited a total of 318 differentially expressed miRNAs (DEMs; 215 upregulated and 103 downregulated) and 647 differentially expressed phasiRNAs (DESs; 521 upregulated and 126 downregulated) (Figs. S4a-c). For example, *miR166*, which targets the *ATHB14* (*HOMEODOMAIN CONTAINING PROTEIN 14*) and *ATHB4* genes (Zhao et al. 2022a; Zhang et al. 2018), was elevated in Hybrid-sh versus both parents but showed no maternal-specific increase in Hybrid-yh (Fig. S4d). On the other hand, the expression of *miR171*, which targets the *GRAS24* and *SCL* (*SCARECROW-LIKE*) genes (Huang et al. 2017; Feng et al. 2023), was upregulated in both hybrids when compared to all parental lines (Fig. S4e). In contrast, the *miR159* expression, targeting *GAMYB2* and *CKX6* (*Cytokinin Oxidase/Dehydrogenase 6*) genes (Zhao et al. 2022b; Jing et al. 2023), showed an opposite trend, with lower levels in the hybrids versus the parents (Fig. S4f). Notably, *miR166*, *miR171*, and *miR159* families dominated hybrid sRNA read counts in F_1_ hybrids (Fig. S4g), while other miRNA families accounted for less than 5% of total reads (Fig. S4h).

### AS-lncRNAs and lincRNAs were reprogrammed in the *Brassica* allotriploid hybrids

**Fig. 2:**
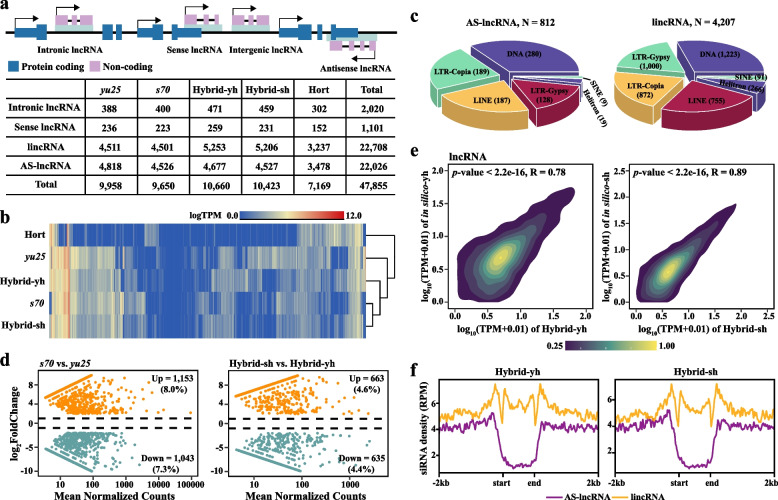
Characterization of lncRNAs in F_1_ hybrids and parental lines. **a** The graph illustrated the classification of lncRNAs into categories such as intronic lncRNA, sense lncRNA, intergenic lncRNA (lincRNA), and antisense lncRNA (AS-lncRNA), along with the number of lncRNAs found in F_1_ hybrids and parental lines.** b** The heat map showed the different expression patterns of lncRNAs in maternal lines (*s70* and *yu25*), paternal line (Hort), and F_1_ hybrids (Hybrid-sh and Hybrid-yh). **c** Number of TE-mediated AS-lncRNA and lincRNA. **d** The MA-plot showed the differentially expressed lncRNAs (DELs) between the maternal lines (left) and between F_1_ hybrids (right). **e** The density plot represented the expression levels of lncRNA in F_1_ hybrids compared to those in silico hybrids, with R indicating Pearson's correlation coefficient. **f** The graph illustrated the distribution of siRNA (RPKM) in AS-lncRNA and lincRNA. **e–f** The Kruskal–Wallis test was employed to determine significant differences

Non-coding RNAs, such as long non-coding RNAs (lncRNAs), regulate diverse cellular processes (Roulé et al. 2022; Wang et al. 2022). We then examined their distribution in the F_1_ hybrids and parental lines to assess hybridization-induced lncRNA activation via strand-specific RNA sequencing (ssRNA-seq). Approximately 20 million clean reads were generated for each sample, with 93.4% mapped to the genome (Table S9). Using established methods (Quan et al. 2022b), we annotated 47,855 lncRNAs in maternal lines, paternal line, and F_1_ hybrids, classifying them as intergenic lncRNAs (lincRNAs, 22,708 loci, 47.5%), antisense lncRNA (AS-lncRNA, 22,026 loci, 46.0%), intronic lncRNA (2,020 loci, 4.2%), and sense lncRNA (1,101 loci, 2.3%) (Fig. 2a). Since less than 6.5% of lncRNA loci were classified as intronic or sense lncRNAs (Fig. 2a), we focused on lincRNAs and AS-lncRNAs expression and function.

A total of 13,346 lncRNAs (including lincRNAs and AS-lncRNAs) were identified in the two F_1_ hybrids, with only 28.3% constitutively expressed (Fig. 2b). In comparison to the parentals, Hybrid-yh and Hybrid-sh retained 4,372 and 4,259 conserved expressed lncRNAs, respectively (Tables S10, S11). A larger proportion of AS-lncRNAs (44%) exhibited conserved expression compared to lincRNAs (23.1%) (Fig. S5a). Additionally, we discovered 535 lncRNAs with specific expressions in Hybrid-yh and 131 in Hybrid-sh (Tables S12, S13). The percentage of lincRNAs uniquely expressed in these hybrids was higher (81% in Hybrid-yh, 67% in Hybrid-sh) than that of AS-lncRNAs (19% in Hybrid-yh, 32% in Hybrid-sh) (Fig. S5b). These findings suggest that AS-lncRNAs exhibit greater expression stability than lincRNAs following interspecific hybridization. Notably, 5,019 lncRNAs were TE-associated, with lincRNAs (4,207) exceedingly AS-lncRNAs (812), DNA-type TEs predominated in both classes, followed by LINE and LTR-Gopia elements (Fig. 2c).

Unlike genes constrained by purifying selection, lncRNAs show dynamic expression (Jackson and Chen 2010). Only 5.8% of genes were differentially expressed across maternal lines, while 15.3% of lncRNAs showed differential expression (Figs. 2d, S5c). This expression pattern was consistently observed in F_1_ hybrids (genes vs. lncRNA = 2.3% vs. 9.0%) (Figs. 2d, S5c), highlighting the significant role of lncRNAs in interspecies transcriptional divergence. Furthermore, the differentially expressed lncRNAs (DE-lncRNA) were predominantly lincRNAs (Fig. S5d), indicating that the lncRNA type greatly influences DE-lncRNA. Two in silico hybrids (in silico-sh and in silico-yh) were then created in a 1:1 ratio of maternal (*s70* and *yu25*) to paternal (*Hort*) RNA-seq data to examine the additive effects of lncRNA expression. The Pearson correlation coefficient (R) between the lncRNA expression of Hybrid-yh and in silico-yh was 0.78, while Hybrid-sh and in silico-sh showed a correlation of 0.89 (Fig. 2e). These correlation values were lower than the 0.86 and 0.95 observed in the genes (Fig. S5e). This suggests that lncRNA expression is more susceptible during interspecific hybridization. siRNAs may modulate lncRNA-DNA interactions via sequence complementarity (Chen & Kim 2024). To investigate whether the distribution of siRNA in lincRNA and AS-lncRNA is biased, we further characterized the distribution of siRNA in these two types of lncRNA. siRNA density was lower in AS-lncRNA bodies compared to flanking regions, while lincRNAs exhibited increased siRNA accumulation in the upstream and downstream 2 kb regions, as well as within their bodies (Kruskal–Wallis, *p* < 2.2e-16) (Fig. 2f), suggesting the lincRNAs are more prone to siRNA targeting, potentially due to sequence motifs enabling siRNA binding.

**Fig. 3:**
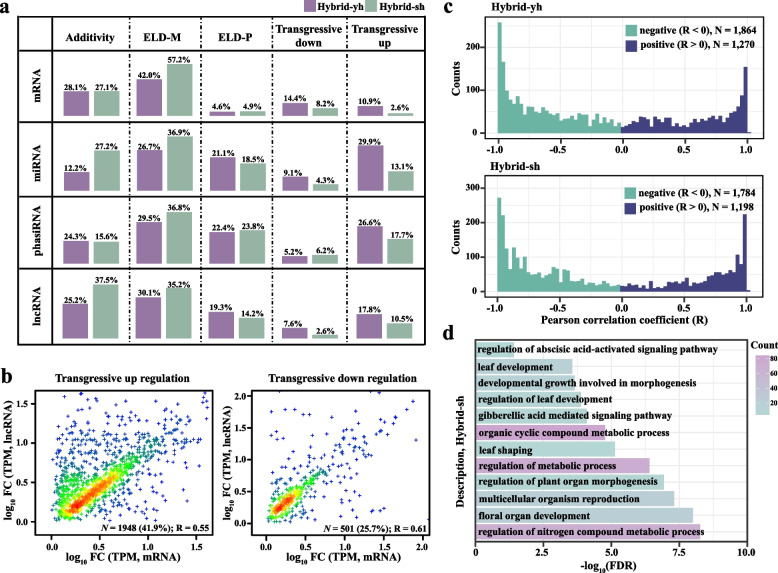
Expression patterns of mRNA and non-coding RNA in F_1_ hybrids. **a** Eight expression patterns were observed for mRNA, miRNA, phasiRNA, and lncRNA. Non-additive expression included two forms: gene expression levels that were similar to one of the parents (expression-level dominance, ELD) and levels that were either lower or higher than the parental levels (transgressive expression). **b** The density plot illustrated transgressively up-regulated lncRNA and *cis* transgressively up-regulated mRNA(left), while transgressively down-regulated lncRNA and *cis* transgressively down-regulated mRNA were shown on the right panel. The Pearson’s correlation coefficient (R) was calculated for this analysis. **c** The graph showed the correlation between miRNA and miRNA target genes. The correlation between miRNA and miRNA target genes was evaluated by Pearson's correlation coefficient (R). A negative correlation was indicated by R < 0. In contrast, a positive correlation was indicated by R > 0. **d** GO enrichment analysis was conducted on miRNA target genes of ELD-M from the Hybrid-sh

### Additive and nonadditive patterns of mRNA and ncRNA

Following polyploid formation, gene expression transitions from dramatic instability to gradual stabilization (Chen 2013). During this process, dynamic shifts in non-additive gene expression — characterized by expression-level dominance (ELD, resembling one parent) or transgressive expression (exceeding parental levels) (Yoo et al. 2014) — reflect genomic shock, epigenetic remodeling, and regulatory network rewiring. We categorized mRNA and ncRNA (miRNA, siRNA, lncRNA) expression in F_1_ hybrids into additive (parental-like) and non-additive patterns. mRNA exhibited consistent additive expression across hybrids (Hybrid-yh: 28.1%; Hybrid-sh: 27.1%), whereas ncRNAs showed genotype-specific additive effects: miRNAs (12.2% vs. 27.2%), phasiRNAs (24.3% vs. 15.6%), and lncRNAs (25.2% vs. 37.5%) in Hybrid-yh and Hybrid-sh, respectively (Fig. 3a). In Hybrid-sh, the levels of ELD-M (maternal-ELD) mRNA and ncRNAs (miRNA, phasiRNA, and lncRNA) were consistently higher than those in Hybrid-yh; in contrast, Hybrid-yh exhibited increased levels of ELD-P (paternal-ELD) miRNA and lncRNA compared to Hybrid-sh, while no significant differences were observed in ELD-P mRNA and siRNA levels between Hybrid-yh and Hybrid-sh (Fig. 3a). Additionally, most of the transgressively regulated mRNAs and non-coding RNAs in Hybrid-yh were greater than those in Hybrid-sh, except for the transgressively downregulated phasiRNAs, where Hybrid-yh showed fewer transgressively downregulated phasiRNAs compared to Hybrid-sh (Fig. 3a). The differences observed between ELD and transgressive across the hybrid combinations (Hybrid-yh vs. Hybrid-sh) indicate a specific interaction among parental genomes.

To assess the regulatory impacts of *cis* (siRNA, lncRNA) and *trans* (miRNA, lncRNA) on the transcriptome, we analyzed transgressively regulated lncRNAs (lincRNAs or AS-lncRNAs, within 5 kb) and phasiRNAs (within 2 kb) on proximal mRNAs (Bartel et al. 2004; Vaucheret et al. 2024; Yu et al. 2022). The results showed that the lncRNAs that were transgressively upregulated exhibited a stronger positive correlation with gene expression (41.9%) compared to the downregulated lncRNAs (25.7%) (Fig. 3b); in contrast, the correlation coefficient between phasiRNAs and adjacent genes was only 0.062 (Fig. S6a). This indicates that lncRNAs significantly enhance mRNA expression, while phasiRNAs have a weaker *cis*-effect on gene expression.

For *trans*-effects, the miRNA information was obtained from the miRNA database miRBase_v22 (Kozomara et al. 2019), and miRNAs targeting mRNAs were predicted using TargetFinder and psRobot (Kiełbasa et al. 2010; Wu et al. 2012), which identified 378 miRNAs targeting 1,408 mRNAs (average of 10 targets per miRNA; Table S14). For instance, *Bna-miRNA-151-5p* and *Bna-miRNA-45-5p* targeted 74 mRNAs, while 48 miRNAs targeted only one gene (Fig. S6b). While the negatively correlated miRNA-mRNA pairs (1,784–1,864) exceeded that of positively correlated pairs (1,198–1,270) (Fig. 3c), with the overall correlation between miRNAs and mRNAs being −0.038 (Fig. S6c). GO enrichment of ELD-M miRNA-targeted mRNAs (346) in Hybrid-yh highlighted responses to stimuli and leaf/organ development (Fig. S6d). In contrast, Hybrid-sh’s targets were associated with gibberellin and abscisic acid pathways (Fig. 3d). Notably, miRNA expression correlated with accessible chromatin regions (ACRs) (Fig. S6e). For example, ELD-M miRNA *Bna-miR169a* had an ACR in its promoter in Hybrid-yh and its maternal *yu25*, aligning chromatin accessibility with miRNA regulation. LncRNAs also influence the expression of nearby genes through *trans*-regulation (Imaduwage and Hewadikaram 2024). We then performed co-expression analyses in all samples using lncRNA and mRNA expression levels (R^2^ > 0.81; target gene > 5 kb), leading to the identification of 1,655 mRNAs trans-regulated by lncRNAs (including lincRNA and AS-lncRNA) (Fig. S6f). These identified lncRNAs were associated with multiple biological processes, such as chromosome localization, root system development, and flavonoid biosynthesis (Fig. S6g).

### ncRNA-mediated DNA methylation had a lesser effect on the expression of neighboring genes in F_1_ hybrids

**Fig. 4:**
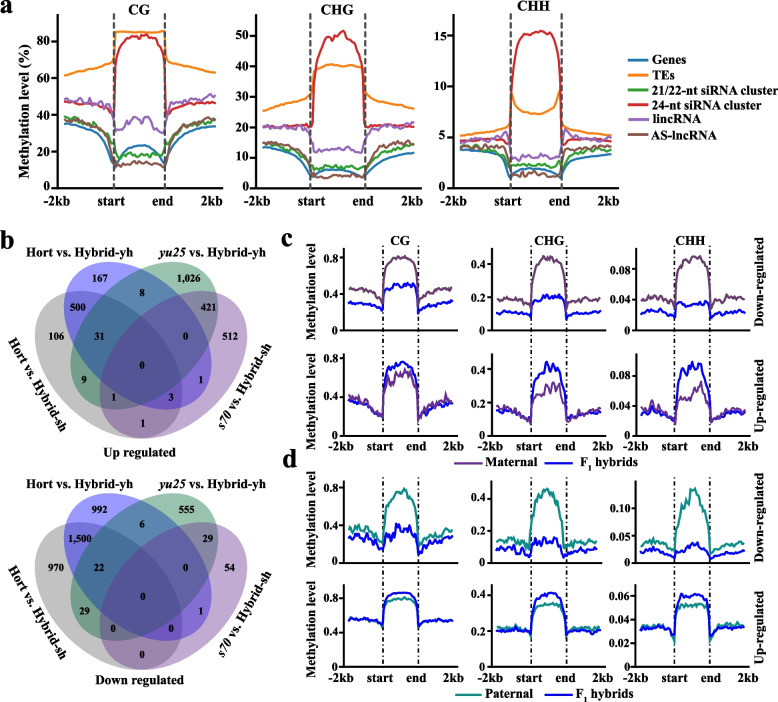
DNA methylation of non-coding RNA in F_1_ Hybrids. **a** Methylation levels of genes, TEs, 21/22-nt siRNA clusters, 24-nt siRNA clusters, Intergenic lncRNAs (lincRNAs) and Antisense lncRNAs (AS-lncRNAs). The Kruskal–Wallis test was employed to determine significant differences (*p* < 0.01). **b** The Venn diagram illustrated the differentially expressed 24-nt siRNA clusters in the Hybrid-sh and Hybrid-yh. **c-d** Compared with the paternal (**c**) and maternal (**d**) lines, DNA methylation levels of differentially up- and down-regulated 24-nt siRNA clusters in F_1_ hybrids. The Kruskal–Wallis test was employed to determine significant differences (*p* < 0.01)

Interspecific hybridization drives DNA methylation dynamics tightly linked to ncRNA expression changes (Ziegler et al. 2023). To investigate ncRNA-methylation relationships, we performed whole-genome bisulfite sequencing (WGBS) with ~ 30 × genome coverage and > 99% bisulfite conversion efficiency across all samples (Table S15). Methylation levels were quantified for genes, transposable elements (TEs), 21/22-nt siRNA clusters, 24-nt siRNA clusters, lincRNAs, and AS-lncRNAs. CG methylation levels of 24-nt siRNA clusters and TEs were comparable but significantly higher than those of lncRNAs (lincRNA/AS-lncRNA), 21/22-nt siRNA clusters, and gene bodies (Kruskal–Wallis, *p* < 2.2e-16) (Fig. 4a). Among all elements, 24-nt siRNA clusters showed the highest CHG and CHH methylation levels, while AS-lncRNA bodies exhibited the lowest (Fig. 4a). Notably, lincRNAs displayed higher methylation than AS-lncRNAs across all contexts (Kruskal–Wallis, *p* < 2.2e-16) (CG, CHG, CHH; Fig. 4a), likely reflecting their elevated siRNA accumulation in lincRNA regions (Fig. 2f), suggesting siRNA density correlates with lincRNA methylation. Furthermore, genes, TEs, siRNA clusters, and lncRNA methylation levels in CHH context tended to be around 0.04 at the upstream and downstream 2 kb regions compared to the CG and CHG contexts (Fig. 4a). We also compared the expression levels of genes adjacent to the 24-nt siRNA cluster, intergenic lncRNA, 21-22nt siRNA cluster, and AS-lncRNA. Genes proximal to 21/22-nt siRNA clusters showed the highest expression levels, whereas those near 24-nt siRNA clusters, lincRNAs, or AS-lncRNAs exhibited no significant differences (Fig. S7a). This suggests that methylation variation in adjacent sequences may not directly drive differential gene expression.

### 24-nt siRNA clusters directly affect DNA methylation levels in F_1_ hybrids

**Fig. 5:**
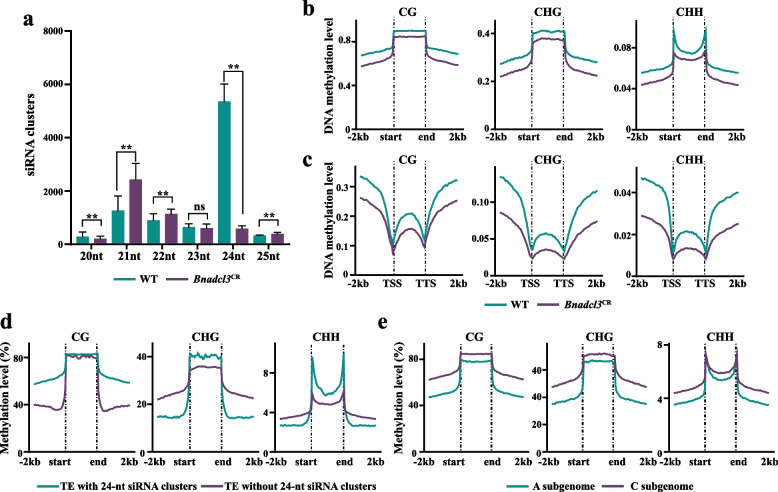
24-nt siRNA cluster-mediated changes in DNA methylation levels. **a** Histograms showing the distribution of siRNA clusters in WT (wild type) and *Bnadcl3*^CR^ mutants. Student’s *t*-test was used to calculate significance (*p* < 0.01). Error bars indicated the mean ± SD of three biological replicates. **b-c** DNA mthylation levels of TEs (**b**) and genes (**c**) in WT and *Bnadcl3*^CR^ mutants. **d** DNA methylation levels of TE with and without 24-nt siRNA clusters. **e** DNA methylation levels of TE in A and C subgenomes of the maternal lines. **b-e** The Kruskal–Wallis test was employed to determine significant differences (*p* < 0.01)

The 24-nt siRNAs guide DNA methylases to TE regions, inducing sequence-specific methylation that suppresses transcriptional activity and transposition frequency, thereby stabilizing the genome (Ziegler et al. 2023). Compared to maternal lines, F_1_ hybrids exhibited more up-regulated than down-regulated 24-nt siRNA clusters, Hybrid-yh notably surpassed Hybrid-sh in the number of differentially expressed 24-nt siRNA clusters (Hybrid-yh: 2,138; Hybrid-sh: 1,024); conversely, compared to paternal line, F_1_ hybrids showed fewer up-regulated than down-regulated 24-nt siRNA clusters (Fig. S7b). In Hybrid-yh and Hybrid-sh, nine and six transgressive expression 24-nt siRNA clusters were identified, respectively (Fig. 4b). Additionally, over 60% of differentially expressed 24-nt clusters were shared between hybrids compared to the paternal lines (Fig. 4b). The F_1_ hybrids displayed elevated DNA methylation at up-regulated 24-nt siRNA clusters compared to parents, while down-regulated clusters showed reduced methylation (Kruskal–Wallis, *p* < 2.2e-16) (Figs. 4c, 4 d), directly linking 24-nt siRNA dynamics to methylation changes. Further, compared to paternal lines, *BnaA06.DCL3* (*Dicer-like 3*), which processes double-stranded RNA (dsRNA) precursors to produce a 24-nt siRNA involved in the RNA-directed DNA methylation pathway (RdDM) (Matzke et al. 2015), was significantly down-regulated in F_1_ hybrids (Student's *t*-test, *p* < 0.01) (Fig. S7c). This suggests that the difference in the number of 24-nt siRNA clusters that are differentially up- and down-regulated compared to the paternal lines may be related to the expression of *BnaA06.DCL3*. Previously, we generated *Bnadcl3*^*CR*^ mutants in *B. napus* (Quan et al. 2025a). In comparison to the wild type (WT), 24-nt siRNA clusters decreased sharply, while 21-nt clusters increased (Student's *t*-test, *p* < 0.01) (Fig. 5a), suggesting compensatory Dicer-like activity (Matzke et al. 2015; Yang et al. 2016). DNA methylation in *Bnadcl3*^*CR*^ was also reduced at gene flanking regions, bodies, and transposable elements (TEs) compared to wild type (Kruskal–Wallis, *p* < 2.2e-16) (Figs. 5b, 5c), confirming *DCL3*’s role in methylation regulation in *B. napus*.

Given that active TEs threaten genome integrity via insertions or rearrangements, 24-nt siRNA-guided TE silencing through methylation is critical for stability (Ziegler et al. 2023). More than 50% of 24-nt siRNA clusters originated from TEs (Fig. S2b). Comparative analysis of TEs with and without 24-nt siRNA clusters revealed significantly elevated CHG and CHH methylation levels in TE bodies associated with these clusters (Kruskal–Wallis, *p* < 2.2e-16) (Fig. 5d), indicating a direct link between 24-nt siRNA clusters and TE hypermethylation. In addition, we investigated the genomic dosage effects by analyzing the maternal line expressed 24-nt siRNA clusters. In the A subgenome, *yu25* and *s70* included 12,236 and 19,209 clusters, while in the C subgenome, they contained 23,970 and 38,389 clusters, respectively (Tables S2, S3). The *B. napus* A and C subgenome size was approximately 374 MB and 542 MB (Song et al. 2020a), respectively, resulting in a genomic size ratio of approximately 2:3. The A: C subgenome 24-nt siRNA cluster ratio was approximately 1: 2, starkly contrasting their genome size ratio (2:3), implying that genome size might not be directly proportional to the number of siRNA clusters. Accordingly, TE methylation levels were significantly higher in the C subgenome (Kruskal–Wallis, *p* < 2.2e-16) (Fig. 5e), suggesting 24-nt siRNAs-mediated RdDM might enhance C subgenome stability.

### ACR-lncRNA regulates gene expression during interspecific hybridization

**Fig. 6:**
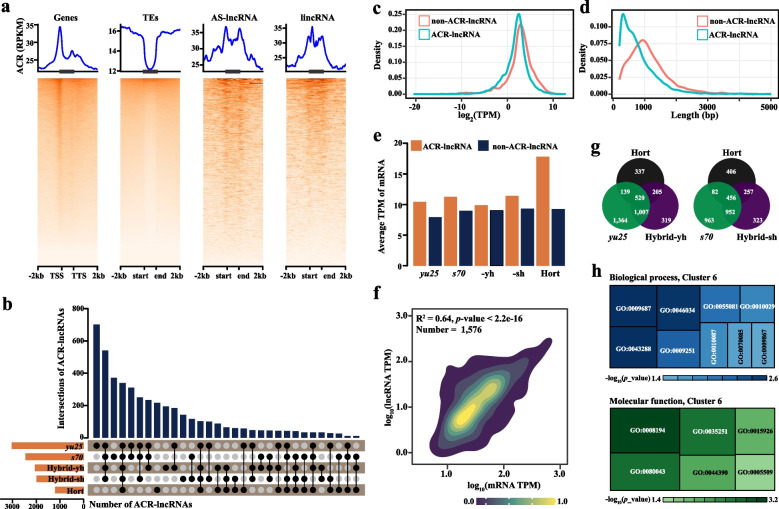
Chromatin enrichment of lncRNA reprogrammed in F_1_ Hybrids. **a** The image displayed chromatin accessibility surrounding genes, transposable elements (TEs), intergenic long non-coding RNAs (lincRNAs), and antisense long non-coding RNAs (AS-lncRNAs). **b** The diagram (upper) and bar graph (bottom) showed the overlap among ACR-lncRNAs and the number of ACR-lncRNA identified in the maternal (*s70* and *yu25*), paternal (Hort), and F_1_ hybrid (Hybrid-sh and Hybrid-yh), respectively. **c**-**d** The density plot showed the gene expression of non-ACR lncRNA and ACR-lncRNA **(c)**, and the transcript length distribution of non-ACR-lncRNA and ACR-lncRNA (**d**). **e** The histogram presented the expression levels of genes located near ACR-lncRNAs compared to those near non-ACR-lncRNAs across all samples. **f** The density plot represented the expression levels of lncRNA compared to mRNA (within 5 kb), with R indicating Pearson's correlation coefficient. **g** The Venn diagram illustrated the proportion of ACR-lncRNAs in F_1_ hybrids relative to their parental lines. **h** Gene Ontology (GO) analysis was conducted on the neighboring genes of ACR-lncRNAs in cluster 6

LncRNAs interact with open chromatin to regulate gene expression by acting as transcription factor cofactors, recruiting chromatin modifiers, or directly binding regulatory elements (Hong et al. 2022; Wang et al. 2022). Using ATAC-seq data from hybrids and parental lines (47 million clean reads per replicate; Table S16) (Quan et al. 2025b), we analyzed accessible chromatin region (ACR) distributions across genes, transposable elements (TEs), and lncRNAs. Both genes and lncRNAs showed distinct transcription start site (TSS) peaks (~ 35 reads), but lncRNA-associated ACRs within gene bodies surpassed mRNA and TE signals (Fig. 6a), suggesting lncRNAs might promote *cis*-regulation via chromatin accessibility during interspecific hybridization.

We identified 3,030 (*yu25*), 2,453 (*s70*), 2,051 (Hybrid-yh), 1,988 (Hybrid-sh), and 1,201 (Hort) ACR-associated lncRNAs (ACR-lncRNAs) (Fig. 6b). Compared to non-ACR-lncRNA, ACR-lncRNA exhibited higher expression levels and longer transcripts (Figs. 6c, d). Additionally, proximal genes near ACR-lncRNAs showed elevated expression (Fig. 6e), with 41.2% of ACR-lncRNAs positively correlating with neighboring genes (Fig. 6f), confirming their *cis*-regulatory role. Furthermore, ACR-lncRNAs were evenly distributed genome-wide compared to bulk ACRs and lncRNAs (Fig. S8), implying broad regulatory influence. Notably, 24.8% (520) of parental and 24.2% (456) of F_1_ hybrid ACR-lncRNAs were chromatin-enriched, while 32.1% and 30.0% of ACR-lncRNAs showed specific enrichment patterns, with 319 (Hybrid-yh) and 323 (Hybrid-sh) novel ACR-lncRNAs emerged (Fig. 6g), highlighting divergent regulatory landscapes between parents and hybrids.

Chromosome exchange and recombination often lead to ACR switching during interspecific hybridization (Li et al. 2022b). A fuzzy *c*-means soft clustering analysis grouped ACR-lncRNAs (including ACR AS-lncRNA and ACR-lincRNA) into six distinct clusters. For example, clusters 1, 2, and 4 represented expression-level dominant ACR-lncRNAs, where the expression levels of ACR-lncRNAs were similar to those of one parent (Fig. S9a). In contrast, cluster 6 indicated transgressive expression of ACR-lncRNAs, where the expression levels exceeded those of the parental lines. ACR-lncRNAs in clusters 3 and 5 displayed more additive expression in F_1_ hybrids than their parental lines (Fig. S9a). Interestingly, Hybrid-yh ACR-lncRNAs dominated clusters 1, 2, 3, and 6, while Hybrid-sh was primarily associated with clusters 2/3 (Fig. S9b). Furthermore, cluster 6 ACR-lncRNAs (725 in Hybrid-yh vs. 453 in Hybrid-sh) neighbored genes involved in glycosylation (e.g., flavonoid 3-O-glucosyltransferase, *UFGT*), apocarotenoid metabolism (e.g., zeaxanthin epoxidase, *ZEP*), and calcium signaling (e.g., *Calmodulin-like protein 45/15*, *CML45/15*) (Fig. 6h), suggesting hybridization-driven metabolic reprogramming.

### ACR-associated AS-lncRNA *lncRNA0410 *enhanced plant drought resistance by positively regulating *BnaA03.MYB12* expression

**Fig. 7:**
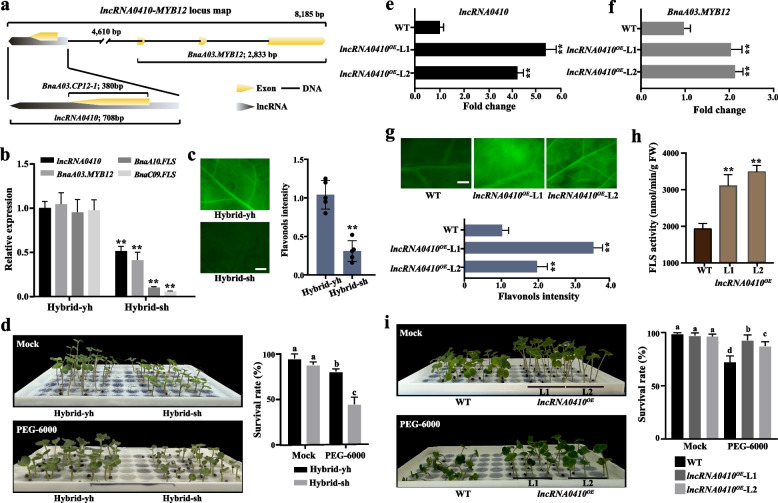
ACR AS-lncRNA *LncRNA0410* enhanced plant drought resistance by positively regulating *BnaA03.MYB12* expression. **a** The graph displayed the gene structure of *lncRNA0410* and *BnaA03.MYB12*. *lncRNA0410*, consisting of a single exon, was transcribed from the antisense strand of *CP12-1*. **b** The graph showed the expression levels of *lncRNA0410*, *BnaA03.MYB12*, *BnaA10.FLS*, and *BnaC09.FLS* in Hybrid-sh and Hybrid-yh. The relative expression levels of each gene were calculated using the 2^−ΔΔCT^ method (Livak & Schmittgen, 2001). The values represented the mean ± SE of three biological replicates, with *BnaActin7* expression used as an internal control. Student's *t*-test was employed to determine significant differences (**, *p* < 0.01). **c** The DPBA staining (left) and quantitation of fluorescence intensity (right) of the Hybrid-sh, and Hybrid-yh leaves. The DPBA fluorescence signal was quantified by ImageJ (https://imagej.net/). The values were the mean ± SE of three biological replicates. Student’s *t*-test was used to calculate significant differences (**, *p* < 0.01). **d** The image (left) showed the seedling growth of Hybrid-sh and Hybrid-yh with or without PEG-6000 (20%) treatment for 10 days. The bar graph (right) showed the survival rate of Hybrid-sh and Hybrid-yh. The values were the mean ± SE of three biological replicates. Statistical test was performed by ANOVA. Different letters indicate significant differences (*p* < 0.01). **e** The graph illustrated the expression levels of *lncRNA0410* in wild-type (WT) and *lncRNA0410*^OE^ lines (T_1_ generation). **f** The graph showed the expression level of *BnaA03.MYB12* in WT (Westar) and *lncRNA0410*^OE^ lines (T_1_ generation). In (**e**) and (**f**), the relative expression level of each gene was calculated using the 2^−ΔΔCT^ method (Livak and Schmittgen, 2001). The values were the mean ± SE of three biological replicates, with *BnaActin7* expression as an internal control. Student’s *t*-test was used to calculate significant differences (**, *p* < 0.01). **g** The DPBA staining (upper) and quantitation of fluorescence intensity (bottom) of the WT (Westar) and *lncRNA0410*^OE^ lines (T_1_ generation) leaves. The DPBA fluorescence signal was quantified by ImageJ (https://imagej.net/). Student’s *t*-test was used to calculate significant differences (**, *p* < 0.01). The values were the mean ± SE of three biological replicates. **h** The graph showed FLS activity in the WT (Westar) and *lncRNA0410*^OE^ lines (T_1_ generation) leaves. Student’s *t*-test was used to calculate significant differences (**,* p* < 0.01). The values were the mean ± SE of three biological replicates. **i** The image (left) and a bar graph (right) showed the survival rate of the WT (Westar) and *lncRNA0410*^OE^ lines (T_1_ generation) with or without 20% PEG-6000 treatment for 10 days. Statistical test was performed by ANOVA. Different letters indicate significant differences (*p* < 0.01). The values were the mean ± SE of three biological replicates

Chromatin-enriched lncRNAs probably act as regulatory elements specific to hybrids, influencing phenotypic traits (Werner et al. 2017). An ACR-mediated AS-lncRNA, *lncRNA0410*, associated with *BnaA03.MYB12,* originated from the 5′ terminal region of the *CP12-1* gene (Fig. 7a). This AS-lncRNA was detected in accessible chromatin regions in the Hybrid-yh but was absent in the Hybrid-sh (Fig. S10a). As expected, the expression level of *BnaA03.MYB12* was markedly reduced in Hybrid-sh relative to Hybrid-yh (Fig. S10b). The alignment results indicated that the *lncRNA0410* genomic sequence was conserved within Brassicaceae but was not found in other plant species (Fig. S11a). In the phylogenetic tree, *lncRNA0410* from *B. napus* clustered closely with *Brassica juncea* and *Brassica carinata*, while differing from *Brassica nigra*, *Brassica rapa*, and *Brassica oleracea* (Fig. S11a). This suggests a potential link between polyploidization and the evolution of *lncRNA0410*. The sequence between *lncRNA0410* and the target gene *BnaA03.MYB12* was then analyzed in eleven *B. napus* genomes, revealing that *lncRNA0410* originated from the sense (e.g., *Express 617*) or antisense (e.g., *Ningyou 7*) strands in different *B. napus* genomes (Fig. S11b).

To investigate whether *lncRNA0410* may regulate the expression of *BnaA03.MYB12*, we analyzed RNA-seq datasets from this study and our previous research (Quan et al. 2022a), conducting a co-expression analysis on six F_1_ hybrids and their respective parental lines. The result revealed a positive correlation with the expression of *BnaA03.MYB12* and *lncRNA0410* (R = 0.65) (Fig. S12a). We then quantified LUC activities by co-transforming *lncRNA0410* with the promoter of *BnaA03.MYB12*. Compared to the control, the LUC activities increased threefold when *lncRNA0410* was co-transformed with the *BnaA03.MYB12* promoter (Fig. S12b). The transcript levels of *BnaA03.MYB12* and its downstream genes, *BnaA10.FLS* and *BnaC09.FLS*, were significantly lower in Hybrid-sh compared to Hybrid-yh (Fig. 7b). Additionally, both flavonol fluorescence signals and FLS enzyme activity were reduced in Hybrid-sh compared to Hybrid-yh (Figs. 7c, S12c).

Under drought stress conditions, plants synthesize a large number of secondary metabolites, such as flavonoids, to enhance their drought resistance (Wang et al. 2021b). We then treated the plants with 20% PEG6000 to mimic the drought stress. Compared to Hybrid-yh (80%), the seedling survival rate decreased for Hybrid-sh (47%) when subjected to the PEG treatment for 10 days (Fig. 7d). We then started to generate the *B. napus lncRNA0410-*overexpressing transgenic lines by transforming the full-length *lncRNA0410* driven by the *35S* Promoter (Fig. S13a). Vectors containing *lncRNA0410* were transferred into the *Agrobacterium tumefaciens* (GV3101), which was used to infect the hypocotyls of *B. napus* cultivar Westar. We obtained six transgenic lines in the T_0_ generation, and Ll and L2 showed the highest expression levels compared to the control and other transgenic lines (Figs. 7e, S13b). We quantified the total flavonol content in WT and two *lncRNA0410*-overexpressing transgenic lines (*lncRNA0410*^*OE*^, Ll and L2, T_0_ generation), and found that the total flavonol content in the *lncRNA0410*^*OE*^ lines was higher than that in the WT (Fig. S13c). In these two *lncRNA0410*^*OE*^ lines (T_1_ generation), the expression of *BnaA03.MYB12* was twice as high as in the control (Fig. 7f). At the seedling stage, the hypocotyl and root lengths of the *lncRNA0410*^*OE*^ lines were longer than those of the wild type (WT) (Fig. S13d). Moreover, the *lncRNA0410*^*OE*^ lines showed increased flavonol fluorescence signals, total flavonol content, and enhanced FLS enzyme activity compared to the WT (Figs. 7g, 7 h, S13e). The survival rate of the *lncRNA0410*^*OE*^ seedling (88% ± 2%) was greater than that of WT (40% ± 7%) following the 20% PEG6000 treatment (Fig. 7i). This suggests that *lncRNA0410* positively regulates *BnaA03.MYB12* expression, thereby enhancing plant drought resistance.

### Dosage-dependent lncRNAs were synergistically affected by siRNA and DNA methylation

**Fig. 8:**
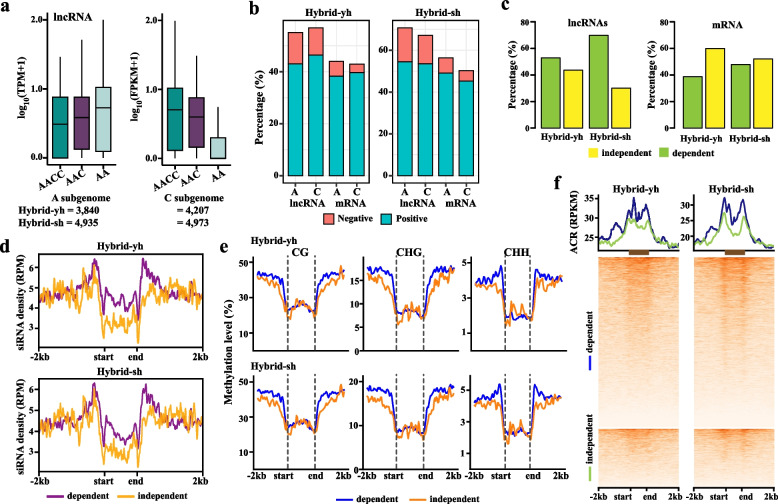
Genome-wide dosage regulation of lncRNA expression in *Brassica* allotriploid. **a** The graph showed the expression level (upper) and number (bottom) of dosage-dependent lncRNAs in the A and C subgenomes. **b** The graph showed the percentage of dosage-dependent genes in negative and positive for lncRNA and mRNA in the A and C subgenomes.** c** The percentage of lncRNAs and mRNAs dosage-dependent and dosage-independent in the Hybrid-sh and Hybrid-yh. **d** The graph showed the distribution of sRNA (RPM) in dosage-dependent and dosage-independent lncRNA in the Hybrid-sh and Hybrid-yh.** e** The graph showed dosage-dependent and dosage-independent lncRNA methylation levels in F_1_ hybrids. **f** The graph showed the chromatin accessibility around dosage-dependent and dosage-independent lncRNA in F_1_ hybrids. **d**-**f** The Kruskal–Wallis test was employed to determine significant differences

Gene dosage balance is critical for normal development and phenotypic stability. Aneuploidy typically induces more pronounced phenotypic disruptions than euploidy, likely due to dosage imbalance (Birchler et al. 2005). LncRNAs exhibit stronger tissue specificity, developmental-stage specificity, and environmental responsiveness than genes (Fort et al. 2021). This suggests that lncRNAs may be more sensitive to variations in genome dosage. To test this, we correlated lncRNA and mRNA expression with A/C subgenome dosage. In the polyploids of AACC (*Brassica napus*), AAC (F_1_ hybrid), and AA (*Brassica rapa*), A-subgenome dosages varied 1/2, 2/3, 1; whereas C-subgenome dosages varied 1/2, 1/3, 0. We then calculated the correlation coefficients (R values) between the expression of lncRNA and mRNA and the relative dosage respectively (Shi et al. 2015; Tan et al. 2016). For example, the Pearson correlation coefficient for A subgenome dosage (1/2:2/3:1) and lncRNA expression values (1.36, 1.76, 5.78) of the *MSTRG.2109.2* was 0.96, with an R^2^ of 0.93, indicating that this lncRNA was dosage-dependent (Fig. S14). In contrast, lncRNAs with R^2^ < 0.64 were classified as dosage-independent lncRNAs (Fig. S14). We identified 3,840 dosage-dependent lncRNAs (dd-lncRNAs) in the A subgenome for Hybrid-yh and 4,935 for Hybrid-sh, while the C subgenome contained 4,207 and 4,973 dd-lncRNAs for Hybrid-yh and Hybrid-sh, respectively (Fig. 8a). Strikingly, 13.1% of dd-lncRNAs negatively correlated with dosage changes, approximately 2.5 times higher than dosage-dependent mRNAs (dd-mRNAs, 5.3%) (Fig. 8b). The dd-lncRNAs also outnumbered dd-mRNAs (56.0–68.9% vs. 39.2–48.0%) (Fig. 8c), confirming lncRNAs’ heightened dosage sensitivity.

Approximately 56% of lincRNAs and 58% of AS-lncRNAs were classified as dd-lncRNAs in Hybrid-yh, whereas 71% of lincRNAs and 72% of AS-lncRNAs were categorized as dd-lncRNAs in Hybrid-sh (Fig. S15a). This suggests genotype-specific dosage responses rather than dependence on lncRNA type. Both dd-lncRNAs and dosage-independent lncRNAs (di-lncRNAs) localized similarly near TEs bodies (Fig. S15b). 6,725 dd-lncRNAs (49.7%), 37,418 dd-mRNAs (56.2%), 2,123 di-lncRNAs (15.8%), and 10,710 di-mRNAs (16.1%) were shared between two F_1_ hybrids (Fig. S15c). These results indicate that the conservation of dosage-independent lncRNAs and mRNAs was weaker than that of dosage-dependent mRNAs and lncRNAs. Furthermore, the conservation level of lncRNAs in the dosage-dependent group was lower than that of mRNAs. The transcriptional regulation of lncRNAs is notably more complex, as they can be influenced by specific transcription factors, epigenetic modifications, and other variables (Wierzbicki et al. 2021).

We further analyzed siRNA, DNA methylation, and accessible chromatin regions (ACRs) regulating lncRNA expression. siRNA was distributed in upstream 2 kb regions of lncRNAs and showed a remarkable correlation with the distance from the lncRNA (dosage-dependent, R = 0.91; dosage-independent, R = 0.84) (Fig. 8d). Additionally, the enrichment of siRNA on lncRNA bodies was significantly greater in dd-lncRNAs than in di-lncRNAs (Kruskal–Wallis, *p* < 2.2e-16; Fig. 8d). We also found that the upstream 2 kb region of the lncRNA was hyper-methylated, whereas the lncRNA bodies was hypo-methylated (Fig. 8e). The dosage-dependent lncRNAs showed significant higher DNA methylation levels at upstream and downstream 2 kb positions than dosage-independent lncRNAs (Kruskal–Wallis, *p* < 2.2e-16; Fig. 8e). Furthermore, enrichment of ACR was detected at upstream and downstream 2 kb regions of lncRNAs, with a greater degree of chromatin opening observed in dd-lncRNAs compared to di-lncRNAs (Kruskal–Wallis, *p* < 2.2e-16; Fig. 8f). These mechanisms—promoter-targeted siRNA/DNA methylation and chromatin opening—work together to regulate lncRNA dosage sensitivity, highlighting their essential role in hybrid gene expression networks.

## Discussion

Interspecific hybridization enhances genetic diversity and drives organismal variety. However, the specific impacts of this process on non-coding RNAs (ncRNAs) remain poorly understood. Clarifying how ncRNAs regulate gene expression is critical to defining their roles in development and evolution. In this study, we generated allotriploid hybrids from *B. napus* and *B. rapa* crosses and analyzed small RNA (sRNA) and long non-coding RNA (lncRNA) dynamics post-hybridization. We observed that ncRNAs interact with DNA methylation and chromatin accessibility to modulate gene expression, ultimately shaping plant growth and adaptation.

### Rapid ncRNA changes after interspecific hybridization affect gene expression

Prior studies suggest that lncRNAs regulate gene expression, buffer genome deletions, and enable epigenetic remodeling in hybrids and polyploids (Kornienko et al. 2023; Palos et al. 2022). Approximately 500 chromatin-enriched lncRNAs are identified in rice, promoting neighboring gene expression via RNA motifs (Zhang et al. 2022). Functional studies show that disrupting these lncRNAs impairs cell dedifferentiation and regeneration (Zhang et al. 2022). We identified 702 transgressive ACR-associated AS-lncRNAs linked to secondary metabolite accumulation and signaling pathways (Fig. 6h). Our work further revealed that the ACR-mediated AS-lncRNA *lncRNA0410* enhances flavonol accumulation and drought tolerance (Fig. 7i). underscoring motif-driven regulatory roles of chromatin-bound lncRNAs.

Hybrid vigor may arise from reshaped gene regulatory networks (Huang et al. 2016; Zhang et al. 2021). We detected rapid sRNA expression shifts post-hybridization, with miRNA families showing divergent inheritance patterns in F_1_ hybrids (Figs. S4d-f). These changes reflect network remodeling, as miRNAs typically repress targets via mRNA complementarity (Zhao et al. 2022a, b). The F_1_ hybrids exhibited negative miRNA-target correlations akin to parents, suggesting altered regulatory patterns could drive novel traits (Li et al. 2021; Ziegler et al. 2023). The *miR166*, *miR171*, and *miR159* families represented about 95% of total reads in F_1_ hybrids. Yet, a high read count for these miRNAs doesn't automatically mean they are crucial regulatory factors; instead, they might only affect the expression of a select few target genes (Tirumalai et al. 2019; Wang et al. 2021a). siRNA presence on lncRNAs implies gene silencing roles, as seen in *Arabidopsis*, where siRNAs target lncRNAs via RNAi to modulate phosphate stress responses (Blein et al. 2020). Additionally, ncRNAs like *IPS1* act as miRNA "sponges," buffering silencing effects on protein-coding genes or stabilizing mRNAs (Franco-Zorrilla et al. 2007)—a compensatory mechanism critical in polyploids and hybrids.

### Non-coding RNAs synergy with epigenetic mechanisms to enhance plant plasticity and adaptability

The interplay between ncRNAs and DNA methylation is essential for epigenetic regulation (Borges et al. 2021; Erdmann et al. 2020). For instance, the lncRNA *COOLAIR* in Arabidopsis modulates *FLOWERING LOCUS C* (*FLC*) expression via DNA methylation, directly influencing flowering time (Zhao et al. 2021). This highlights ncRNAs’ ability to fine-tune gene expression through epigenetic mechanisms. A reciprocal feedback loop further links DNA methylation and ncRNA activity: methylation shapes ncRNA expression, while ncRNAs conversely regulate methylation patterns (Satyaki et al. 2019; Erdmann et al. 2020). This dynamic relationship maintains precise control over gene activation and silencing. For example, 24-nt siRNAs guide DNA methylation of transposable elements (TEs) through RNA-directed pathways, ensuring genomic stability (Li et al. 2022a). Following interspecific hybridization, we observed rapid remodeling of ACR-associated lncRNAs, with over 30% exhibiting distinct enrichment patterns (Fig. 6b). These chromatin-accessible regions—often promoters or enhancers—are critical regulatory hubs. Studies across plant species confirm that ncRNAs interact with chromatin states to influence gene activity (Zhang et al. 2022). In rice, lncRNAs linked to chromatin changes during seed development suggest roles in coordinating growth-related genes (Wang et al. 2018). These findings underscore ncRNAs’ capacity to orchestrate plant development by dynamically modulating chromatin architecture. In polyploid plants, elevated dosage of specific ncRNA genes can disrupt target gene regulation, ultimately altering phenotypic outcomes (Martinez et al. 2018). Such dosage sensitivity modulates gene expression by influencing chromatin structure, DNA methylation, or RNA stability. The gene balance theory posits that precise stoichiometry among dosage-sensitive gene groups is critical for maintaining normal physiological functions (Borges et al. 2018). ncRNAs play a central role in preserving this equilibrium, particularly by regulating dosage-sensitive protein-coding genes. They achieve this through direct interactions with mRNA or proteins, fine-tuning their abundance or activity. In hybrid or polyploid systems, ncRNA dosage effects intersect with environmental cues, shifts in regulatory networks, and expression changes in interacting genes. These synergies often deviate from predicted ncRNA dosage outcomes, driving adaptive or plastic gene expression responses (Borges et al. 2018; Martinez et al. 2018).

### The overall size of the transcriptome is nonlinear to gene expression

The gene balance hypothesis posits that selective pressure maintains stoichiometric equilibrium among proteins within dosage-sensitive complexes by regulating gene copy numbers (Papp et al. 2003; Birchler et al. 2012). This implies that gene dosage determines protein abundance and coordinates expression across interacting genes. However, empirical data on dosage effects remain limited, and existing studies reveal significant variability in transcriptional responses (Coate et al. 2016). To evaluate transcriptome scaling and dosage effects following ploidy changes, we sequenced transcriptomes of synthetic *Brassica* allotriploid alongside their diploid and tetraploid progenitors. Consistent with earlier reports (Tan et al. 2016; Shi et al. 2015), genome duplication does not proportionally double transcriptome size. Additionally, gene dosage responses varied markedly across ploidy levels, indicating a weak correlation between gene expression and genomic copy number.

In *Arabidopsis*, the scaling of the mRNA transcriptome does not align proportionally with genome doubling or halving, as most genes do not display a 1:1 dosage-response relationship (Song et al. 2020b). Similar inconsistencies with linear dosage effects are observed in maize and soybean (Guo et al. 1996; Coate et al. 2010), paralleling our findings. The transcriptional responses to dosage alterations in *Arabidopsis* remain nonlinear, even with small-scale duplications (SSD) (Song et al. 2020b). Importantly, this nonlinearity occurs regardless of how the dosage is modified—through allele deletion/duplication, SSD, or whole-genome duplication (WGD)—with the majority of genes deviating from the anticipated 1:1 ratio between genomic dosage and transcript abundance (Song et al. 2020b; Hou et al. 2018). Additionally, transcriptional responses to WGD differ significantly across various polyploidization events (Hou et al. 2018; Yu et al. 2010), likely due to *cis*-regulatory mechanisms, epigenetic changes, and transposable element activity (Xiao et al. 2022; Shi et al. 2015). Together, these elements elucidate why WGD does not consistently increase transcript levels for all genes.

## Materials and methods

### Plant materials

*Brassica napus* ‘*s70*’ and ‘*yu25*’ (*B. napus L*.; AACC, allotetraploid) were selected as the maternal parents, while *Brassica rapa* species (*B. campestris L. ssp. chinensis var. purpuria* Hort.; AA, diploid) served as the paternal parent. The F_1_ allotriploid hybrids were generated through a cross between two different *B. napus* varieties and the *B. rapa* species. All plant materials were grown under the same field conditions at Huazhong Agricultural University (30°28ʹN, 114°21ʹW). Stem epidermal tissue from below the fifth true leaf at the top from different parents and F_1_ hybrids was collected on the same day (10:00—11:00 am in December, with an average temperature of 8 °C) to prevent environmental effects. The collected samples were immediately frozen in liquid nitrogen. Three biological replicates were obtained for each sample from 3–5 individual plants. These samples were subsequently used for Strand-Specific RNA sequencing (ssRNA-seq), small RNA sequencing (sRNA-seq), Assay of Transposase Accessible Chromatin sequencing (ATAC-seq), Whole-Genome Bisulfite Sequencing (WBGS) library construction, and metabolite analysis.

### Constructs and transgenic lines

The full-length DNA sequence (708 bp) of *lncRNA0410* was amplified by the primers forward primer (5’-ATCCATCGATAGTACTGTCGACAAATAAACATCTTCGCGTAACCGG-3’) and reverse primer (5’-TCCTCGCCCTTGCTCACCATGGTACCAAATCGTGATCACAGAGTTCTGTATTTATCT-3’), and the resultant DNA fragment was sub-cloned into *pCAMBIA1300-mCherry-flag* vector, resulting in construct *p35S*:*lncRNA0410*-*mCherry-flag*. The positive construct was introduced into *B. napus* cultivar Westar by *Agrobacterium tumefaciens*-mediated transformation using hypocotyl as materials (Tang et al. 2018). Briefly, *B. napus* hypocotyl explants were incubated with *Agrobacterium* infection buffer (recipe) for 20 min and then transferred to M1 media [Sucrose (30.0 g L^−1^), Mannitol (18.0 g L^−1^), 2,4-D (1.0 mg L^−1^), Kinetin (0.3 mg L^−1^), Acetosyringone (100.0 µM), Agarose (6.0 g L^−1^), pH = 5.8] plates in the dark for 48 h. The explants were then transferred to M2 media [Sucrose (30 g L^−1^), Mannitol (18 g L^−1^), Agarose, 2,4-D (1.0 mg L^−1^), Kinetin (0.3 mg L^−1^), Timentin (300 mg L^−1^), Kanamycin (50 mg mL^−1^), pH = 5.8] plates with appropriate selection antibiotics to induce callus growth. The calluses were transferred to M3 [Glucose (10 g L^−1^), Xylose (0.25 g L^−1^), MES (0.6 g L^−1^), Zeatin (2 mg L^−1^), IAA (0.1 mg L^−1^), Timentin (300 mg L^−1^), Kanamycin (25 mg L^−1^), *p*H = 5.8], followed by M4 media (Agarose and Sucrose, *p*H = 5.8), to allow shoot and root regeneration, respectively. RT-qPCR confirmed the positive transgenic plants by using primers forward primer (5’-GCACCATACGAACCAAACCAAA-3’) and reverse primer (5’-TCCTTTGGAAGAATACTGCAGTG-3’). The transgenic lines were named as *lncRNA0410*^OE^. All plants were grown in a greenhouse under a 100 μmol m^−2^ s^−1^ light intensity with a 16/8 h light/dark photoperiod at 22 °C. The primers used for vector construction were listed in Table S17.

### Strand-specific RNA sequencing analysis

For RNA-seq analysis, Trimmomatic_v0.38 (java -jar trimmomatic-0.38.jar PE -threads 4 -phred33 TruSeq3-PE.fa:2:30:10 SLIDINGWINDOW:4:15 MINLEN:36 LEADING:3 TRAILING:3) was utilized to remove barcode adaptors and low-quality reads (Bolger et al. 2014). Subsequently, the filtered reads were aligned to the *B. napus* (Zhongshuang 11, *ZS11*) reference genome using HISAT2_v2.2.0 (hisat2 -p 8 -x) with default parameters (Kim et al. 2015; Song et al. 2020a). The uniquely mapped reads were filtered using SAMtools (Li et al. 2009). Transcripts Per Million (TPM) was conducted on BAM files with StringTie_v2.1.4 (stringtie -e -G -o) (Pertea et al. 2015). Genes with TPM values greater than 1 were considered as expressed genes. Those genes meeting the criteria of an adjusted *p*-value < 0.05 by DESeq2_v1.36.0 and a |log_2_fold change|≥ 1.5 was identified as differentially expressed genes (Love et al. 2014).

### The lncRNAs identification pipeline

To identify high-confidence lncRNAs, the transcripts underwent rigorous filtration steps. Initially, transcripts from each dataset were assembled separately using the StringTie_v2.1.4 program (Pertea et al. 2015). Subsequently, all transcripts from each species were merged to create final transcripts using StringTie –merge. These newly assembled transcripts were compared with reference genome annotations using gffcompare (Pertea, G. and Pertea, M. 2015). Transcripts with class codes 'i,' 'u,' 'o,' and 'x' were retained, indicating full containment within reference introns, intergenic sequences, and antisense sequences of known genes. Candidate transcripts were further selected based on coding potential scores derived from annotation programs CNCI_v2.0 (python CNCI.py -f lnc01 -p 10 -m pl) (Sun et al. 2013), CPC_v2.0 beta (python CPC2.py -i) (Kong et al. 2007), and PLEK_v1.2 (python PLEK.py -fasta -minlength 200) (Li et al. 2014b). Transcripts with scores of 0 were excluded. HMMER_v3.3 eliminated transcripts containing known protein domains (Finn et al. 2011). Transcripts longer than 200 nucleotides were retained, while those with maximal TPM < 0.5 in samples were eliminated to identify high-confidence, expressed lncRNAs. Constitutively expressed lncRNAs were defined as those lncRNAs present in all samples (TPM > 0.5). Accessible chromatin regions associated with lncRNAs (ACR-lncRNAs) were defined based on criteria from previous studies (Werner et al. 2017; Zhang et al. 2022), where the lncRNAs overlap the sequences of accessible chromatin regions by more than 50%, and at least two replicated lncRNAs in biological replicates contained ACRs. Novel ACR-lncRNAs: ACR-lncRNAs were identified in F_1_ hybrids (TPM > 0.5) but not in their parents (TPM < 0.1) (Kornienko et al. 2023).

### Small RNA sequencing analysis

The raw sequencing reads were trimmed using cutadapt to remove adapters (https://github.com/ marcelm/cutadapt/tree/v3.1) (cutadapt -a AGATCGGAAGAGCACACGTCT -m 18 -M 30 –discard-untrimmed). Subsequently, sRNAs between 18 and 30-nt in length were selected and mapped to the *B. napus* (ZS11) genome using bowtie_v1.1.2 (bowtie -v 0 -m 1 –best –strata -x) and defined into siRNA clusters using Shortstack_v3.8.4 (ShortStack –readfile –bowtie_cores 5 –sort_mem 10G –mismatches 0 –ranmax 5 –mincov 1 rpm –dicermin 20 –dicermax 26) (Johnson et al. 2016). The Novel miRNA and PhasiRNA was predicted using a pipeline centered on miRDeep-P2_v1.1.4 (bash miRDP2-v1.1.4_pipeline.bash -g -f -L 15 -M 0 -R 1 -p 1 -o) (Kuang et al. 2019) (http://www.mirbase.org/) and PHASIS_v3.3 (https://github.com/atulkakrana/PHASIS/wiki). sRNA-mapped reads were normalized to the total cleaned reads for further analysis. The sRNAs with an adjusted *p*-value < 0.05 identified by DESeq2 and a |log2fold change|≥ 1.0 were characterized as differentially expressed sRNA.

### ATAC-seq analysis

The low-quality reads and adapters from the raw ATAC-seq data were filtered and removed using Trimmomatic (Bolger et al. 2014). The clean data were then aligned to the *B. napus* (*ZS11*) reference genome by Bowtie2_v2.5.2 (bowtie2 -p 8 -q -I 10 -X 1000 –dovetail –no-unal –very-sensitive-local –no-mixed –no-discordant -x) (Langmead & Salzberg 2012; Song et al. 2020a). The mapped reads in sam format were converted to bam format using SAMtools_v1.9 (samtools view -b -f 2 -q 30 -o) (Li et al. 2009). Subsequently, MACS2_v2.2 (macs2 callpeak -t -n -g –nomodel –shift −100 –extsize 200) peak calling software was used to identify ATAC-seq peaks (Zhang et al. 2008). The overlapping peaks over 50 bp in the biological replicates were considered ACRs. The genomic distribution of ACRs and associated genes was confirmed using the ChIPseeker_v1.32.1 tool (Yu et al. 2015). Differential binding events were identified using the DiffBind_v3.6.5 package (Ross-Innes et al. 2012). The motifs of the ACRs were identified using HOMER_v5.1 (Heinz et al. 2010).

### WGBS analysis

We used BatMeth2_v.2.01 (BatMeth2 pipel –region 200 bp –binCover 4 –Qual 5 −1 −2 -g -p 20 -o –gff) with default parameters to map the filtered WGBS reads to the *B. napus* (ZS11) genome. The sequences covering five or more cytosine sites were set as valid methylation sites. To identify the differentially methylated regions (DMRs), the whole genome was divided into 200-bp bins. Each bin contained at least five cytosines. Only cytosine regions with adjusted *p*-values < 0.01 and DNA methylation differences greater than 0.3, 0.2, and 0.1 (for CG, CHG, and CHH, respectively) were considered DMRs (BatMeth2 batDMR -g -o_dm -o_dmr −1 −2) (Zhou et al. 2019). BatMeth2-Meth2BigWig generated BigWig files to identify and visualize DMRs in IGV.

### Dosage-dependent analysis

The identification of dosage-dependent and -independent mRNA and lncRNA was followed in the previous studies (Shi et al. 2015; Tan et al. 2016). Briefly, Pearson correlation tests, employing the Benjamini and Hochberg false discovery rate (FDR) method, were conducted to assess the relationship between single transcript expression and the relative dosage for the A subgenome (1/2:2/3:1) and C subgenome (1/2:1/3:0). Transcript exhibiting a significant correlation between expression and dosage (R^2^ > 0.64, FDR < 0.05) were classified as dosage-dependent. In contrast, transcript with R^2^ < 0.64 were deemed dosage-independent. For instance, the Pearson correlation coefficient for A subgenome dosage (1/2:2/3:1) and lncRNA expression values (1.36, 1.76, 5.78) of the *MSTRG.2109.2* was 0.96, with an R^2^ of 0.93, indicating that this gene was dosage-dependent.

### Functional enrichment analysis

The function descriptions of genes were acquired from the *B. napus* (ZS11) reference genome (Song et al. 2020a). The GO enrichment analysis was performed using agriGO2, and terms with an FDR < 0.05 were considered significant.

### Dual-luciferase assay

The full length of *lncRNA0410* was amplified by PCR and inserted into the plant binary vector pGreenII-62SK for transient overexpression. The 2-kb promoter sequence of *BnaA03.MYB12* was amplified by PCR and inserted into the pGreenII 0800-LUC vector using a ClonExpress II One Step Cloning Kit (C112, Vazyme, China). Then, all relative effector and reporter constructs were transformed into Arabidopsis mesophyll protoplasts as described previously (Huang et al. 2015). After that, the dual luciferase assay was performed according to the instructions of the Dual-Luciferase® Reporter Assay System (E1910, Promega, WI, USA). Data were expressed as the ratio of firefly to ranila luciferase activity (Fluc/Rluc). Each data point included at least three replicates, and three independent experiments were conducted for each experiment. The primers used were listed in Table S18.

### DPBA staining

From each material, 20–30 high-quality seeds were selected, soaked in 70% ethanol for 30 s, and rinsed with ddH_2_O for 3 min. These seeds were subsequently planted in a hydroponic box for 20 days. The DPBA staining was performed as previously described (Stracke et al. 2010). From each material, 20–30 high-quality seeds were selected, soaked in 70% ethanol for 30 s, and rinsed with ddH_2_O for 3 min. These seeds were subsequently planted in a hydroponic box for 20 days. The 2-3th leaves of plants were bleached with ethanol overnight at room temperature and stained with a freshly prepared 0.25% (w/v) diphenyl boric acid 2-aminoethyl ester (DPBA) for at least 20 min. The fluorescence was visualized on a fluorescence microscope (Ax10, Zeiss, Berlin, Germany) with an excitation wavelength of 340–380 nm and a 425 nm emission wavelength, and the gray value was calculated using ImageJ_v1.8.0 (Schneider et al. 2012). For comparative analyses, average flavonol signals in control leaves were set as 1. The experiment was independently replicated three times.

### Total flavonol quantification

Total flavonol quantification was performed following the methodology described by Tirumalai et al. (2019). From each material, 20–30 high-quality seeds were selected, soaked in 70% ethanol for 30 s, and rinsed with ddH_2_O for 3 min. These seeds were subsequently planted in a hydroponic box for 20 days. Approximately 500 mg leaf tissue were then collected and ground into a fine powder using liquid nitrogen and extracted with 10 mL of 75% ethanol for 3 h. The filtrate was dried and re-dissolved in 5 mL of 75% ethanol. 0.1 mL of this solution was transferred to a clear 2 mL tube, by adding 0.5 mL of sodium nitrite (50 mg ml⁻^1^) and mixing vigorously, followed by 6 min incubation. Subsequently, 0.5 mL of aluminum nitrate was added to the reaction mixture, which was then incubated for an additional 6 min. The reaction was terminated by adding 2.5 mL of 1 M sodium hydroxide, and the absorbance was measured at 510 nm after a 15-min incubation period. A standard curve was created using varying concentrations of quercetin, ranging from 0 to 100 µg, to quantify the amount of quercetin produced in the samples based on absorbance measurements. For each replicate, three independent quantifications were performed for each sample, and the experiment was repeated three times independently.

### PEG treatment

From each material, 20–30 high-quality seeds were selected, soaked in 70% ethanol for 30 s, and rinsed with ddH_2_O for 3 min. These seeds were subsequently planted in a hydroponic box. Following 3 days of germination, stress experiments were conducted under control (0% PEG-6000) and drought (20% PEG-6000) conditions, with three replicas for each treatment.

### FLS activity assay

Flavonoid synthase (FLS) activity was determined using the FLS activity kit (MEIAO, MO-Z15804, China). A 0.1 g leaf sample was collected, ground in a mortar with liquid nitrogen, and homogenized with 1 mL of extract solution in an ice bath. The resulting crude enzyme extract was centrifuged at 4 °C and 8000 rpm for 10 min. 10 µL of the extract was transferred to a labeled plate, followed by the addition of 40 µL of the sample dilution solution and 100 µL of the horseradish peroxidase (HRP)-labeled detection antibody. The mixture was then mixed thoroughly and incubated in a water bath at 37 °C for 60 min. The absorbance (OD value) at a wavelength of 450 nm was quantified using a spectrometer (Spark, Tecan, Switzerland). For each replicate, three independent quantifications were performed for each sample, and the experiment was repeated three times independently.

### RT-qPCR assay

The quantitative reverse transcription-PCR (RT-qPCR) assay was conducted following the methodology outlined in Quan et al. (2022a). The melting curve analysis of RT-qPCR confirmed the presence of a single peak. Gene expression levels were determined using the 2^−ΔΔCT^ method and each analysis was replicated a minimum of three times. *BnaActin7* (XM_013858992) genes served as internal controls, and the primer details can be found in Table S19.

### Statistics and reproducibility

Statistical significance was assessed with R (https://r-project.org). The Kruskal–Wallis test and ANOVA were conducted using the *kruskal.test* and *aov* functions (*p* < 0.01) from the R package.

## Supplementary Information

Supplementary Material 1. Figure S1. Schematic model of the diploid species (*B. rapa*, Hort), F_1_hybrids, and the allotetraploid species (*B. napus*, *s70,* and *yu25*). Figure S2. The distribution of siRNA clusters in F_1_ hybrids and parental lines. Figure S3. Identification of phasiRNAs in F_1_ hybrids and parental lines. Figure S4. The differentially expressed sRNA in F_1_ hybrids and parental lines. Figure S5. The reprogrammed lincRNA and AS-lncRNA in F_1_ hybrids. Figure S6. The correlation between phasiRNA and miRNA and their target genes. Figure S7. Differentially expressed ncRNAs in F_1_ hybrids. Figure S8. Distribution of genes, ACR, non-ACR lncRNA, and ACR-lncRNA in F_1_ hybrids. Figure S9. Expression patterns of ACR-lincRNAs and ACR-AS-lncRNAs. Figure S10. The ACR and expression of* lncRNA0410 *and *BnaA03.MYB12* in F_1_ hybrids. Figure S11. Evolution of* lncRNA0410 *in Brassicaceae species. Figure S12. *lncRNA0410* positively regulated the expression of *BnaA03.MYB12*. Figure S13. Construction and phenotypic identification of *lncRNA0410*-overexpressing transgenic lines. Figure S14. Identification of dosage-dependent and dosage-independent lncRNAs. Figure S15. Genome-wide dosage regulation of the expression of lncRNAs in F_1_ hybrids.

Supplementary Material 2. Table S1. Statistics of small RNA-seq data and reads mapping for all samples. Table S2. The list of sRNA clusters in maternal *yu25*. Table S3. The list of sRNA clusters in maternal *s70*. Table S4. The list of sRNA clusters in Hybrid-yh. Table S5. The list of sRNA clusters in Hybrid-sh. Table S6. The list of sRNA clusters in paternal Hort. Table S7. The list of identified miRNAs. Table S8. The list of identified phasiRNAs. Table S9. Statistics of ssRNA-seq data and reads mapping for all samples. Table S10. The number of conservatively expressed lncRNA in Hybrid-yh. Table S11. The number of conservatively expressed lncRNA in Hybrid-sh. Table S12. The number of specifically expressed lncRNA in Hybrid-yh. Table S13. The number of specifically expressed lncRNA in Hybrid-sh. Table S14. The number of miRNA-targeted mRNAs was predicted using TargetFinder and psRobot software. Table S15. Statistics of WGBS data and reads mapping for all samples. Table S16. Statistics of ATAC-seq data and reads mapping for all samples. Table S17. Primer sequences used for overexpressing transgenic lines. Table S18. Primer sequences used for Dual-luciferase analyses. Table S19. Primer sequences used for RT-qPCR analyses.

## Data Availability

All data supporting this study's findings are included within the article and its online Supplementary Materials. All high-throughput sequencing data for RNA-seq, small RNA-seq, ATAC-seq, and WGBS of Brassica allotriploid hybrids and their parents in this study are available in the Short Read Archive (SRA) under NCBI BioProject accession number PRJNA1154317. The raw data can also be queried at https://gigadb.org/dataset/102668.

## Abbreviations

ACRs: Accessible Chromatin Regions
ATAC-seq: Assay for Transposase-Accessible Chromatin with high throughput sequencing
DMRs: Differentially methylated regions
FDR: False discovery rate
TPM: Transcripts Per Million
LUC: Luciferase
PCA: Principal component analysis
RNA-seq: RNA sequencing
RT-qPCR: Reverse Transcription quantitative Polymerase Chain Reaction
small RNA-seq: sRNA sequencing
TE: Transposable element
TSS: Transcription start sites
TTS: Transcription termination site
WGBS: Whole Genome Bisulfite Sequencing
