# Supplementary material for: Genome-wide reprogramming of sRNA and lncRNA in the epigenetic regulation following interspecific hybridization in the Brassica species
Source: Mol Hortic. 2026 Feb 11;6:14. doi: 10.1186/s43897-025-00194-8 (PMC12892563; doi:10.1186/s43897-025-00194-8)
Supplement: Supplementary file 1 — Supplementary Material 1. Figure S1. Schematic model of the diploid species (B. rapa, Hort), F1hybrids, and the allotetraploid species (B. napus, s70, and yu25). Figure S2. The distribution of siRNA clusters in F1 hybrids and parental lines. Figure S3. Identification of phasiRNAs in F1 hybrids and parental lines. Figure S4. The differentially expressed sRNA in F1 hybrids and parental lines. Figure S5. The reprogrammed lincRNA and AS-lncRNA in F1 hybrids. Figure S6. The correlation between phasiRNA and miRNA and their target genes. Figure S7. Differentially expressed ncRNAs in F1 hybrids. Figure S8. Distribution of genes, ACR, non-ACR lncRNA, and ACR-lncRNA in F1 hybrids. Figure S9. Expression patterns of ACR-lincRNAs and ACR-AS-lncRNAs. Figure S10. The ACR and expression of lncRNA0410 and BnaA03.MYB12 in F1 hybrids. Figure S11. Evolution of lncRNA0410 in Brassicaceae species. Figure S12. lncRNA0410 positively regulated the expression of BnaA03.MYB12. Figure S13. Construction and phenotypic identification of lncRNA0410-overexpressing transgenic lines. Figure S14. Identification of dosage-dependent and dosage-independent lncRNAs. Figure S15. Genome-wide dosage regulation of the expression of lncRNAs in F1 hybrids. [file 43897_2025_194_MOESM1_ESM.docx]

**Title Page**

**Genome-wide reprogramming of sRNA and lncRNA in the epigenetic regulation after interspecific hybridization in the *Brassica* species**

Chengtao Quan^1,2^, Ling Wang^1,2^, Hangpan Wei^1,2^, Yilin Ma^1,2^, Yunqin Wang^1,2^, Guoting Cheng^3^, Chaozhi Ma^1,2^, and Cheng Dai^1,2^

1 National Key Laboratory of Crop Genetic Improvement, Huazhong Agricultural University, Wuhan 430070, China

2 Hubei Hongshan Laboratory, Wuhan, 430070, China

3 College of Informatics, Huazhong Agricultural University, Wuhan 430070, China.

The author responsible for the distribution of materials integral to the findings presented in this article following the policy described in the Instructions for Authors is:

Cheng Dai (cdai@mail.hzau.edu.cn)

To whom correspondence should be addressed.

Dr. Cheng Dai

National Key Laboratory of Crop Genetic Improvement, Huazhong Agricultural University, Wuhan 430070, P.R. China

Email: [cdai@mail.hzau.edu.cn](mailto:cdai@mail.hzau.edu.cn)

**Supporting Information**

**Supplemental materials and methods**

ATAC-seq Experiment

WGBS Experiment

ssRNA-seq and sRNA-seq Experiment

**Supplemental figure legends**

**Figure S1.** Schematic model of the diploid species (*B. rapa*, Hort), F_1_ hybrids, and the allotetraploid species (*B. napus*, *s70,* and *yu25*).

**Figure S2.** The distribution of siRNA clusters in F_1_ hybrids and parental lines.

**Figure S3.** Identification of phasiRNAs in F_1_ hybrids and parental lines.

**Figure S4.** The differentially expressed sRNA in F_1_ hybrids and parental lines.

**Figure S5.** The reprogrammed lincRNA and AS-lncRNA in F_1_ hybrids.

**Figure S6.** The correlation between phasiRNA and miRNA and their target genes.

**Figure S7.** Differentially expressed ncRNAs in F_1_ hybrids.

**Figure S8.** Distribution of genes, ACR, non-ACR lncRNA, and ACR-lncRNA in F_1_ hybrids.

**Figure S9.** Expression patterns of ACR-lincRNAs and ACR-AS-lncRNAs.

**Figure S10.** The ACR and expression of *lncRNA0410* and *BnaA03.MYB12* in F_1_ hybrids.

**Figure S11.** Evolution of *lncRNA0410* in Brassicaceae species.

**Figure S12.** *lncRNA0410* positively regulated the expression of *BnaA03.MYB12*.

**Figure S13.** Construction and phenotypic identification of *lncRNA0410*-overexpressing transgenic lines.

**Figure S14.** Identification of dosage-dependent and dosage-independent lncRNAs.

**Figure S15.** Genome-wide dosage regulation of the expression of lncRNAs in F_1_ hybrids.

**Supplemental Tables**

**Table S1.** Statistics of small RNA-seq data and reads mapping for all samples.

**Table S2.** The list of sRNA clusters in maternal *yu25*.

**Table S3.** The list of sRNA clusters in maternal *s70*.

**Table S4.** The list of sRNA clusters in Hybrid-yh.

**Table S5.** The list of sRNA clusters in Hybrid-sh.

**Table S6.** The list of sRNA clusters in paternal Hort.

**Table S7.** The list of identified miRNAs.

**Table S8.** The list of identified phasiRNAs.

**Table S9.** Statistics of ssRNA-seq data and reads mapping for all samples.

**Table S10.** The number of conservatively expressed lncRNA in Hybrid-yh.

**Table S11.** The number of conservatively expressed lncRNA in Hybrid-sh.

**Table S12.** The number of specifically expressed lncRNA in Hybrid-yh.

**Table S13.** The number of specifically expressed lncRNA in Hybrid-sh.

**Table S14.** The number of miRNA-targeted mRNAs was predicted using TargetFinder and psRobot software.

**Table S15.** Statistics of WGBS data and reads mapping for all samples.

**Table S16.** Statistics of ATAC-seq data and reads mapping for all samples.

**Table S17.** Primer sequences used for overexpressing transgenic lines.

**Table S18.** Primer sequences used for Dual-luciferase analyses.

**Table S19.** Primer sequences used for RT-qPCR analyses.

**Supplemental materials and methods**

**ATAC-seq Experiment**

For each biological replicate, the collected plant tissue was cut into small pieces with blade in 500 mL lysis buffer (15 mM Tris-HCl pH7.5, 20 mM NaCl, 80 mM KCl, 0.5 mM spermidine, 5 mM 2-mercaptoethanol and 0.2% Triton X-100). After confirming nuclear integrity, purified nuclei were resuspended in a 50 μL Tn5 transposase integration reaction and incubated at 37°C for 30 min. The Tn5 transposase-digested DNA fragments were then recovered using a MinElute PCR Purification Kit (Qiagen, Cat No. 28004), and followed by purification and amplification. The purified library was then sequenced on an Illumina Novaseq platform by Novogene Gene Technology (Novogene, Beijing, China). All ATAC-seq profiles were generated from at least of three independent biological replicates.

**WGBS Experiment**

Genomic DNA was extracted from 10 samples using the cetyl trimethylammonium bromide (CTAB) method. A lambda DNA spike-in was utilized to correct for non-conversion rates of uracil, with 1 ng of methyl-free lambda DNA added to 1 μg genomic DNA as an internal reference for the conversion test. Bisulfite conversion of DNA was carried out using the EZ DNA Methylation Gold Kit (Zymo Research in Irvine, California, USA). The Bisulfite-Seq Library Prep Kit for Illumina (Novogene in Beijing, China) was used to construct whole genome bisulfite sequencing (WGBS) libraries, which were then sequenced on an Illumina HiSeq X10 platform at a depth of 30-fold. Two biological replicates were performed.

**RNA-seq and small RNA-seq Experiment**

According to the manufacturer's instructions, total RNA was extracted with RNeasy Plant Mini Kit (Qiagen, Cat No./ID: 74904). This preparation was split and was used for both RNA-seq and sRNA-seq. According to the manufacturer's protocols, library construction and deep sequencing were performed using the Illumina HiSeq 4000 Platform (Novogene, Beijing, China). Two or three biological replicates were performed.

**Supplemental figure legends**


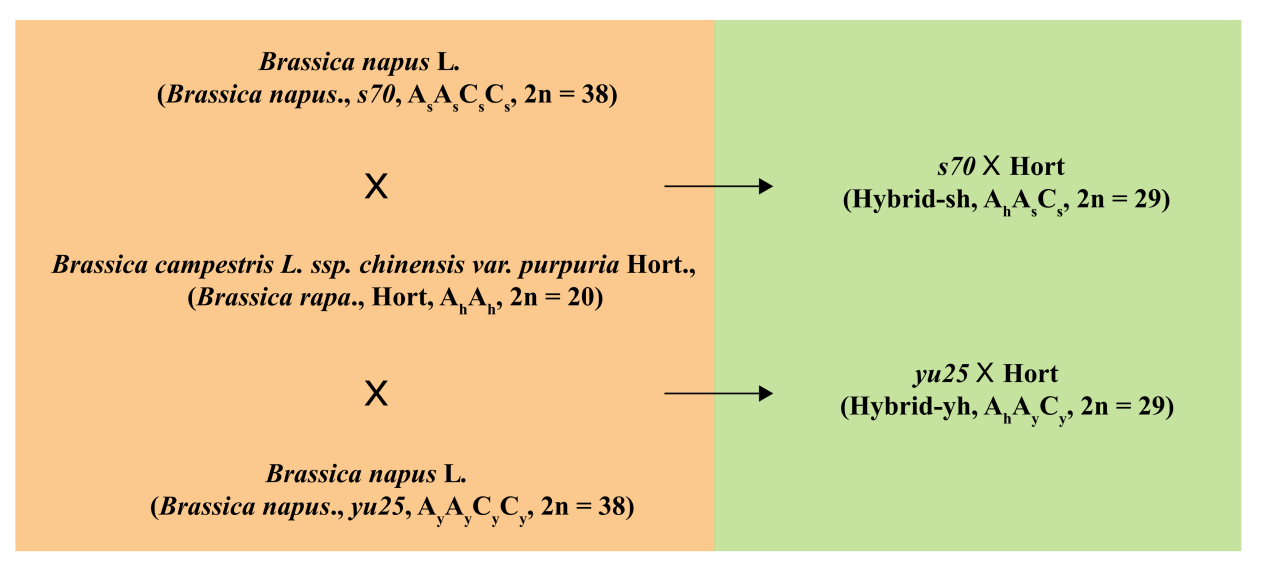


**Figure S1. Schematic model for the diploid species (*B. rapa*, Hort), F_1_ hybrids, and the allotetraploid species (*B. napus*, *s70* and *yu25*).**

Two allotriploid *Brassica* species hybrids were generated by crossing two *B. napus* inbred lines (s*70*, A_s_A_s_C_s_C_s_; *yu25*, A_y_A_y_C_y_C_y_) with a *B. rapa* line (Hort, A_h_A_h_), resulting in the hybrids Hybrid-sh (*s70* × Hort, A_s_A_h_C_s_) and Hybrid-yh (*yu25* × Hort, A_y_A_h_C_y_), respectively.


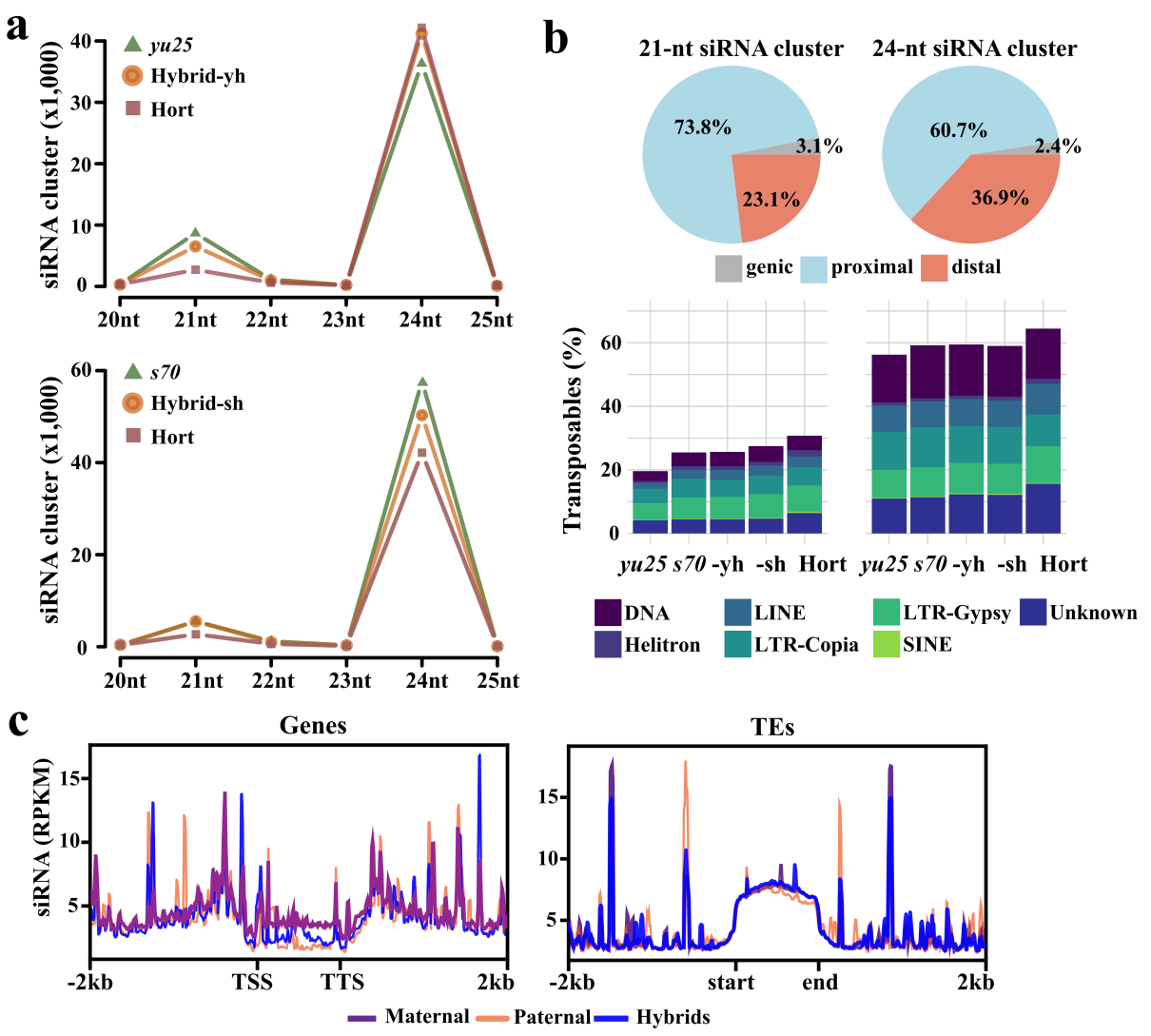


**Figure S2. The distribution of siRNA clusters in F_1_ hybrids and parental lines.**

**(a)** Histograms showed the number of siRNA clusters in F_1_ hybrids and their parental lines. **(b)** The pie charts showing the distribution of 21-nt and 24-nt siRNA clusters among various genomic features. These features were categorized as genic (overlapping with a gene), proximal (within 2 kb of a gene), distal (more than 2 kb away from any gene), and transposable elements (TE). The suffixes -sh and -yh refer to Hybrid-sh and Hybrid-yh, respectively. **(c)** 24-nt siRNA clusters distribution of genes (left) and TEs (right) in parental lines and F_1_ hybrids. The Kruskal-Wallis test was employed to determine significant differences (*p* < 0.01).


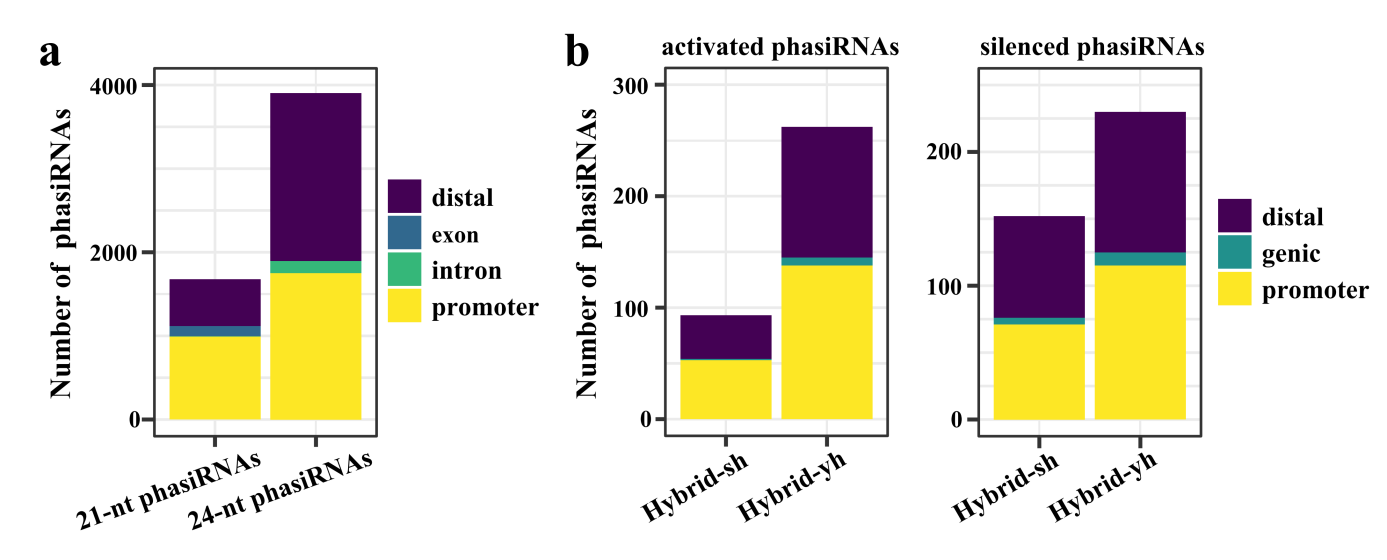


**Figure S3. Identification of phasiRNAs in F_1_ hybrids and parental lines.**

**(a)** The number of 21-nt and 24-nt phasiRNAs in all samples. **(b)** The number of activated phasiRNAs and silenced phasiRNAs in F_1_ hybrids.


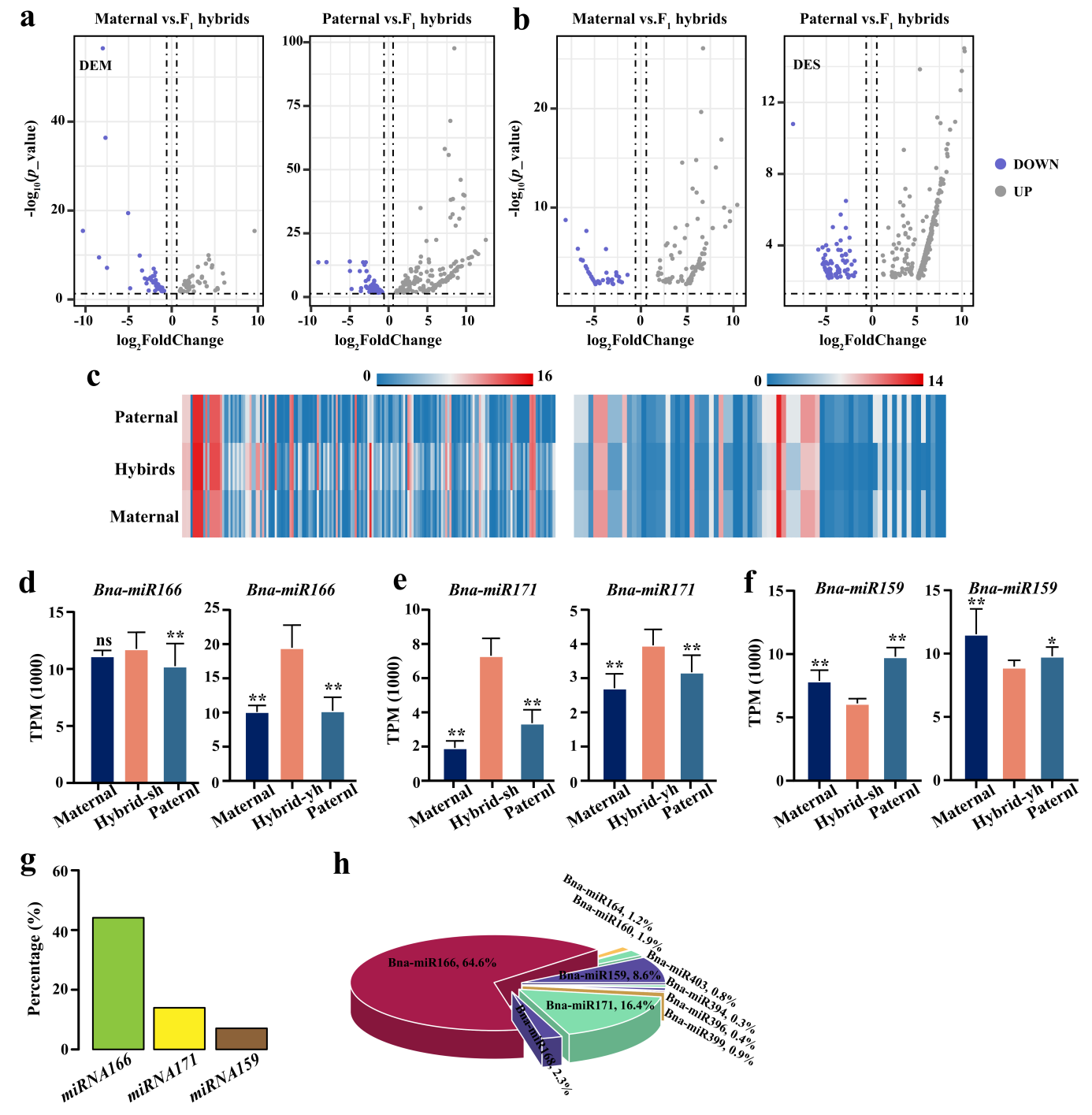


**Figure S4. The differentially expressed sRNA in F_1_ hybrids and parental lines.**

**(a) - (b)** The graphs showed the number of differentially expressed miRNA (DEM) **(a)** and differentially expressed phasiRNA (DES) (**b**). Data points meeting the *p*_value < 0.05 and log_2_FC > 1.0 criteria were in grey, while those with *p*_value < 0.05 and log_2_FC < -1 were in blue. **(c)** Heat map represents differentially up-regulated (left) and down-regulated (right) miRNAs compared with parents. **(d)** - **(f)** The expression of *Bna-miR166* **(d)**, *Bna-miR171* **(e)**, and *Bna-miR159* **(f)** in hybrids and their parents. miRNAs with an adjusted *p*-value < 0.05 identified by DESeq2 and a |log2fold change| ≥ 1.0 were assigned as differentially expressed; **p* < 0.05, ***p* < 0.01. Error bars indicated means ±SD from three biological replicates. **(g)** The percentage of reads of *miR166*, *miR171* and *miR159* families in the total reads. **(h)** The pie chart showed the top 10 miRNAs with the largest proportion of reads.


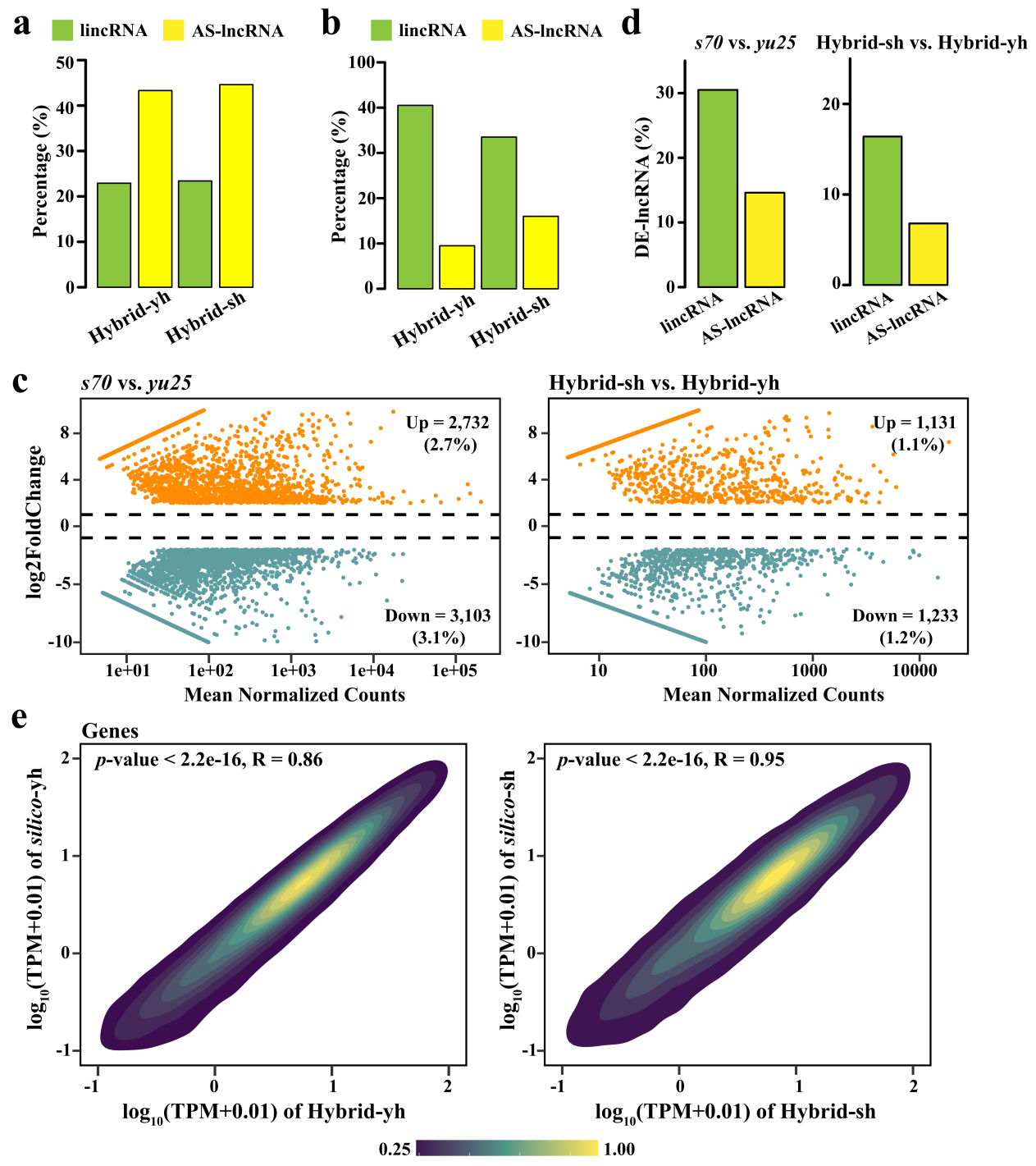


**Figure S5. The reprogrammed lincRNA and AS-lncRNA in F_1_ hybrids.**

**(a)** The percentage of conservatively expressed lincRNA and AS-lncRNA in Hybrid-sh and Hybrid-yh. **(b)** The percentage of specifically expressed lincRNA and AS-lncRNA in Hybrid-sh and Hybrid-yh. **(c)** MA-plot showed differentially expressed genes (DEGs) between maternal lines (left) and between F_1_ hybrids (right). **(d)** The percentage of differentially expressed lincRNAs and AS-lncRNAs for both the maternal lines (left) and F_1_ hybrids (right). **(e)** Density plot representing the expression of gene in F_1_ hybrids compared to *in* *silico* hybrids. R is the Pearson's correlation coefficient. The Kruskal-Wallis test was employed to determine significant differences.


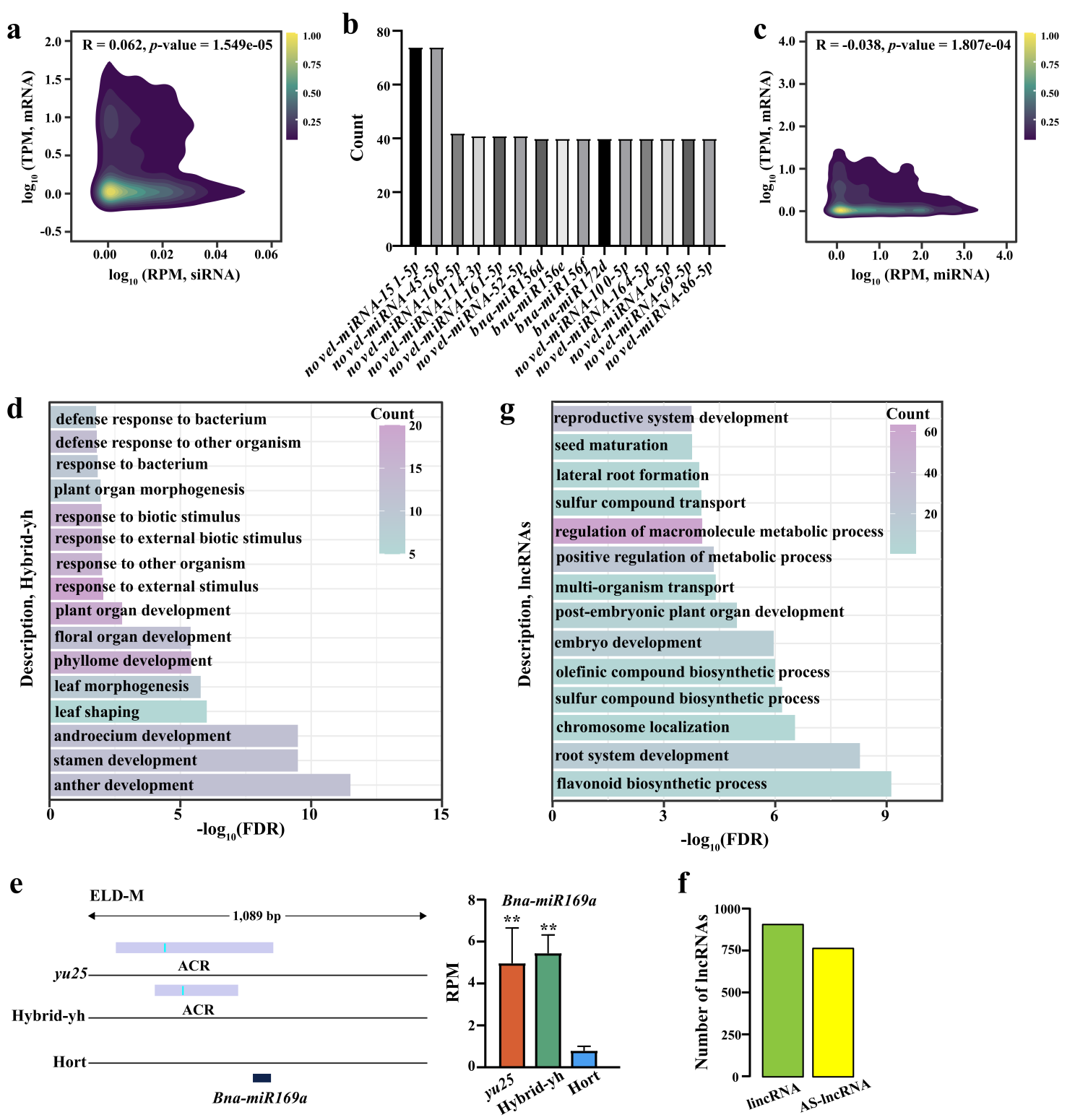


**Figure S6. The correlation between phasiRNA and miRNA and their target genes.**

**(a)** Positive correlation (R = Pearson’s correlation coefficient) of expression level of siRNA (x-axis) with proximal mRNA (y-axis). The proximal mRNA was located within 2 kb of the siRNA. **(b)** The bar graph showed the number of genes targeted by the top 10 miRNAs. **(c)** Negative correlation (R = Pearson’s correlation coefcient) of expression level of miRNA (x-axis) with target mRNA (y-axis). **(a)**, **(c)**, the Kruskal-Wallis test was employed to determine significant differences. **(d)** GO enrichment analysis was conducted on miRNA target genes of ELD-M from the Hybrid-yh. **(e)** Genome browser showed ACR around *Bna-miR169a* in maternal *s70*, paternal Hort, and Hybrid-sh (left) and the expression levels of *Bna-miR169a* in maternal *s70*, paternal Hort, and Hybrid-sh (right). Error bars indicate means ±SD from three biological replicates. Student’s *t*-test; ***p* < 0.01. **(f)** The number of lncRNAs with *trans*-regulation of mRNAs. **(g)** GO enrichment analysis was conducted for lncRNAs *trans*-regulated mRNAs.


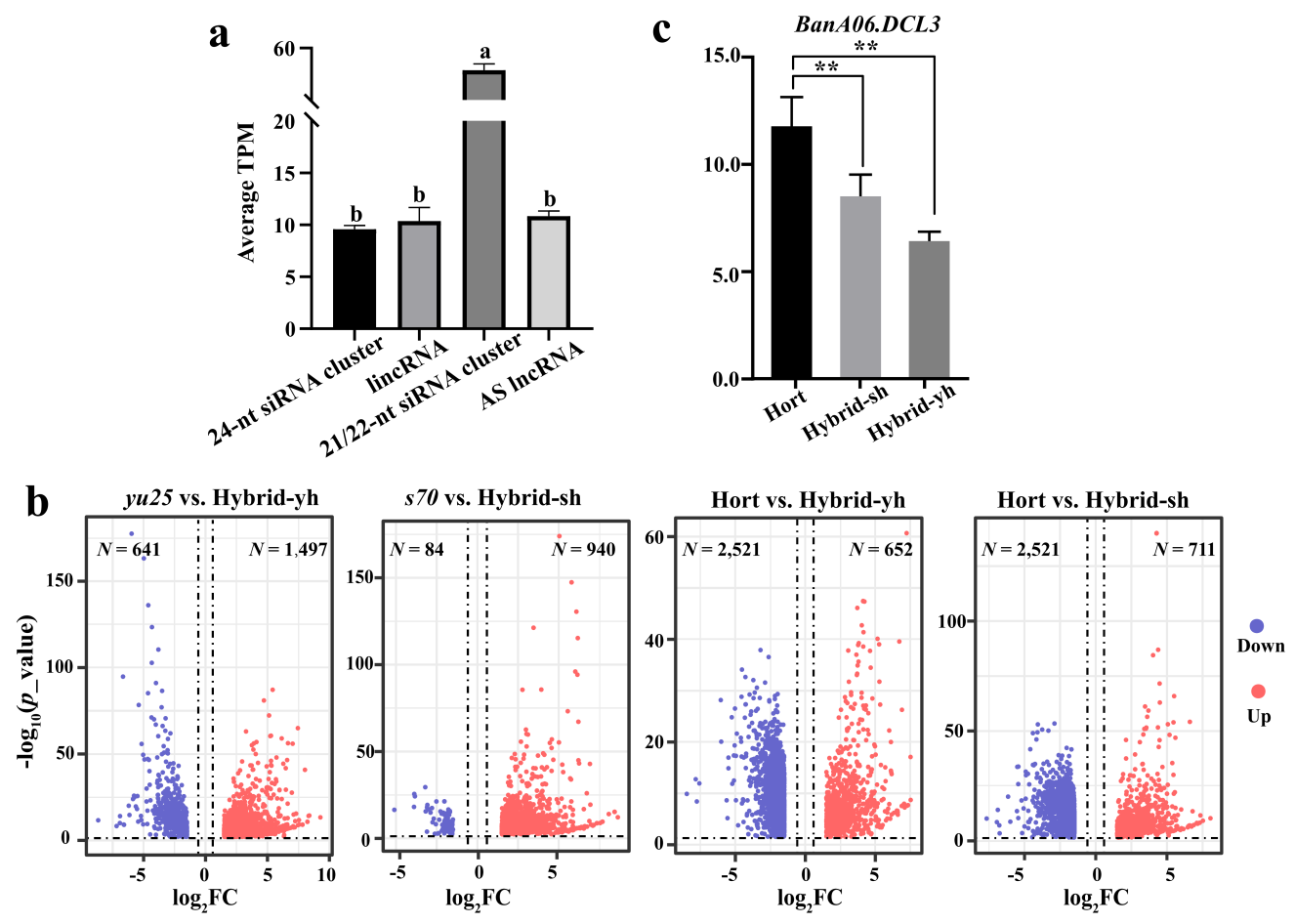


**Figure S7. Differentially expressed ncRNAs in F_1_ hybrids.**

**(a)** Expression levels of genes located near 24-nt sRNA clusters, lincRNA, 21/22-nt sRNA clusters, and AS-lcnRNA. ANOVA performed statistical tests. Different letters indicate significant differences (*p* < 0.01). Error bars indicate means ±SD from three biological replicates. **(b)** The graphs showed the number of differentially expressed 24-nt sRNA clusters in F_1_ hybrids compared to maternal and paternal lines. 24-nt sRNA clusters with an adjusted *p*-value < 0.05 identified by DESeq2 and a |log_2_fold change| ≥ 1.0 were assigned as differentially expressed. **(c)** The expression of *BnaA06.DCL3* in Hybrid-sh, Hybrid-yh, and paternal Hort. Genes with an adjusted *p*-value < 0.05 identified by DESeq2 and a |log_2_fold change| ≥ 1.0 were assigned as differentially expressed; ***p* < 0.01. Error bars indicate means ±SD from three biological replicates.


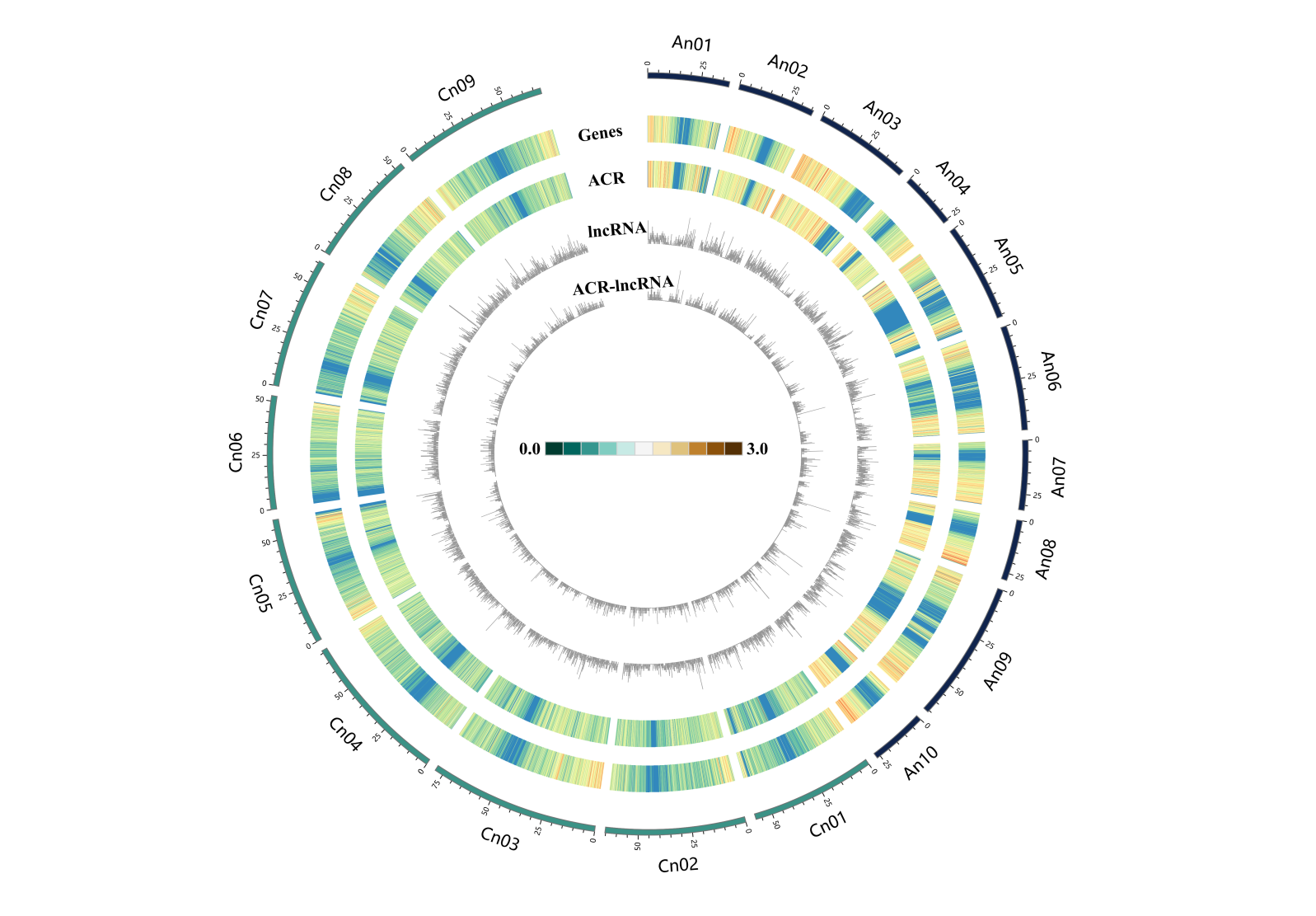


**Figure S8. Distribution of ACR, non-ACR lncRNA, and ACR-lncRNA in F_1_ hybrids.**

Browser Circos plots showed the genome distribution of genes, ACRs, non-ACR lncRNAs, and ACR-lncRNAs regions.

**
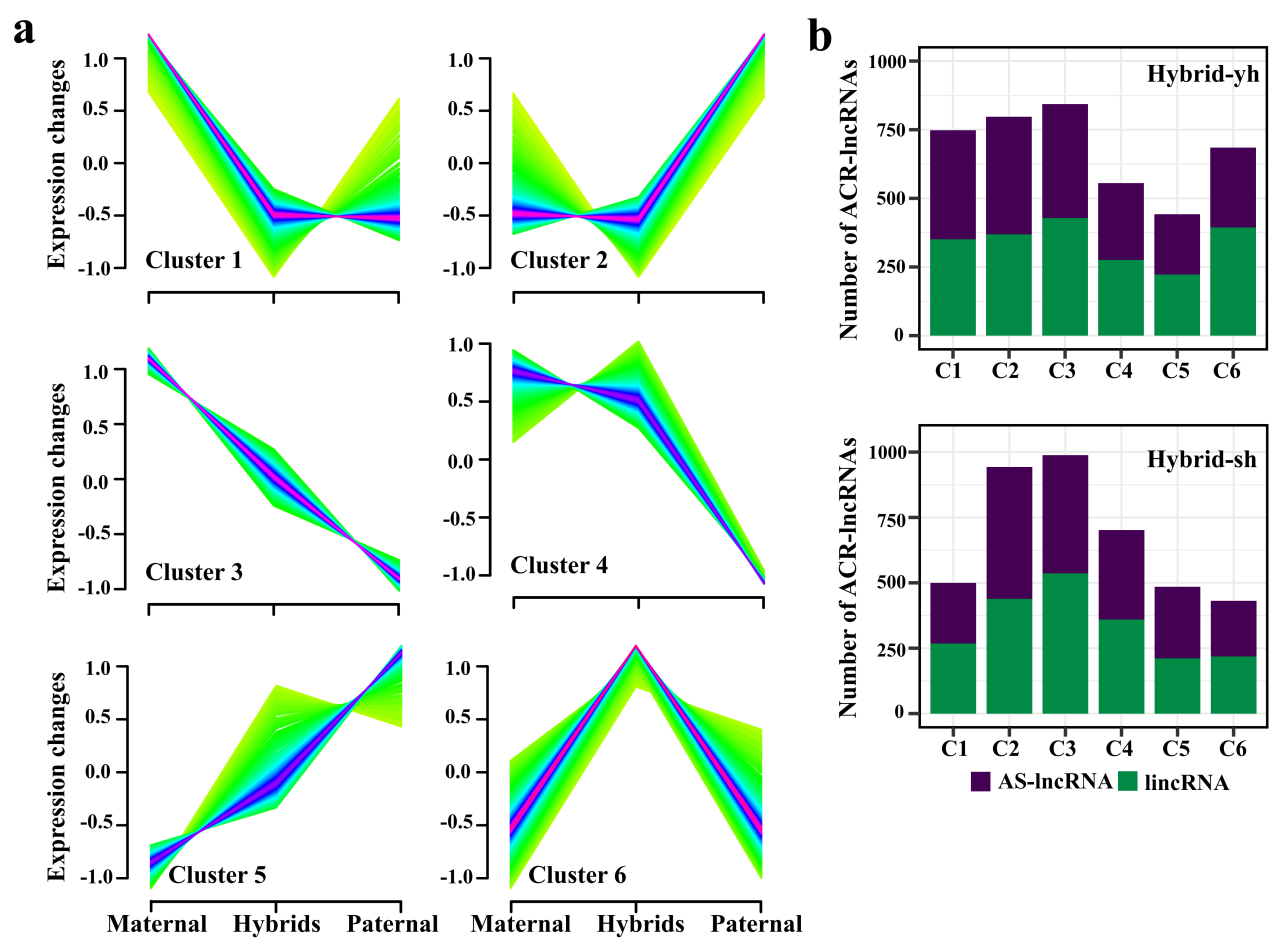
**

**Figure S9. Expression patterns of ACR-lincRNAs and ACR-AS-lncRNAs.**

**(a)** The graphs showed the *c*-means soft clustering analysis of the chromatin accessibility levels of lncRNAs in F_1_ hybrids and their parental lines. (**b**) The bar plot showed the number of ACR-AS-lncRNA and ACR lincRNA of each cluster of Hybrid-yh (upper) and Hybrid-sh (bottom).


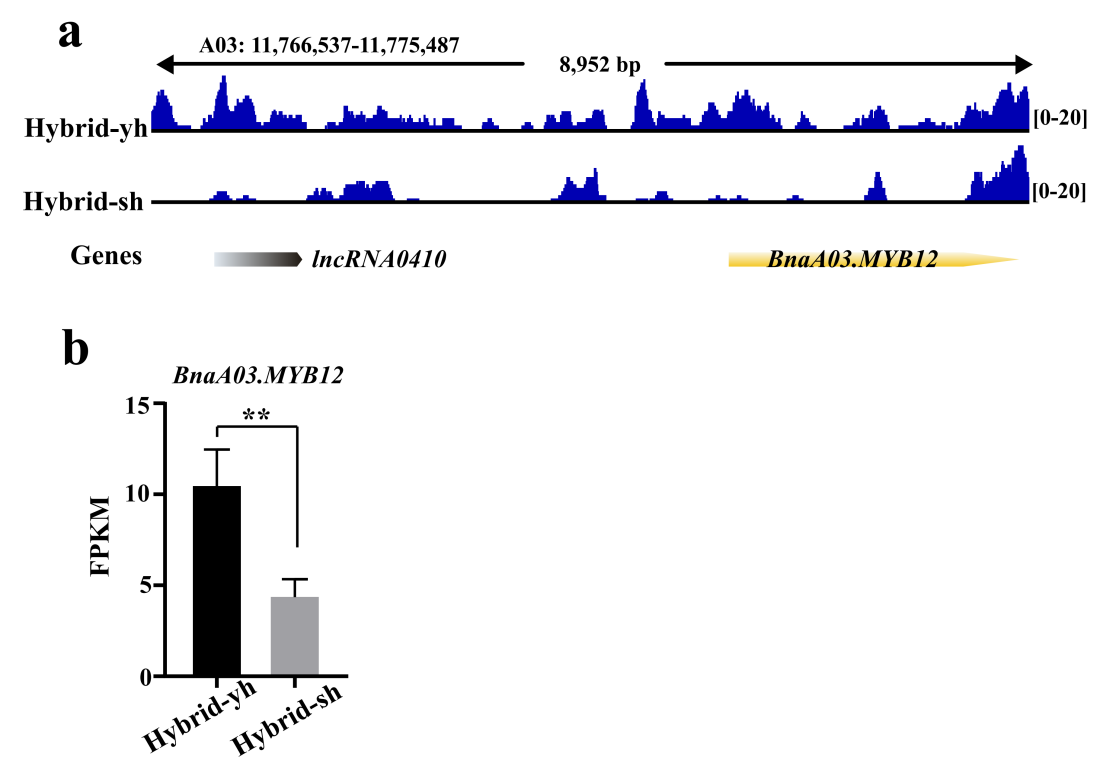


**Figure S10. The ACR and expression of *lncRNA0410* and *BnaA03.MYB12* in F_1_ hybrids.**

**(a)** The genome browser displayed ATAC-seq signals surrounding *lncRNA0410* and *BnaA03.MYB12* in both Hybrid-sh and Hybrid-yh. **(b)** The graph showed the gene expression of *BnaA03.MYB12* in Hybrid-sh and Hybrid-yh. Error bars indicated the mean ±SD of three biological replicates. Student’s *t*-test was used to calculate significance where ** indicated *p* < 0.01.


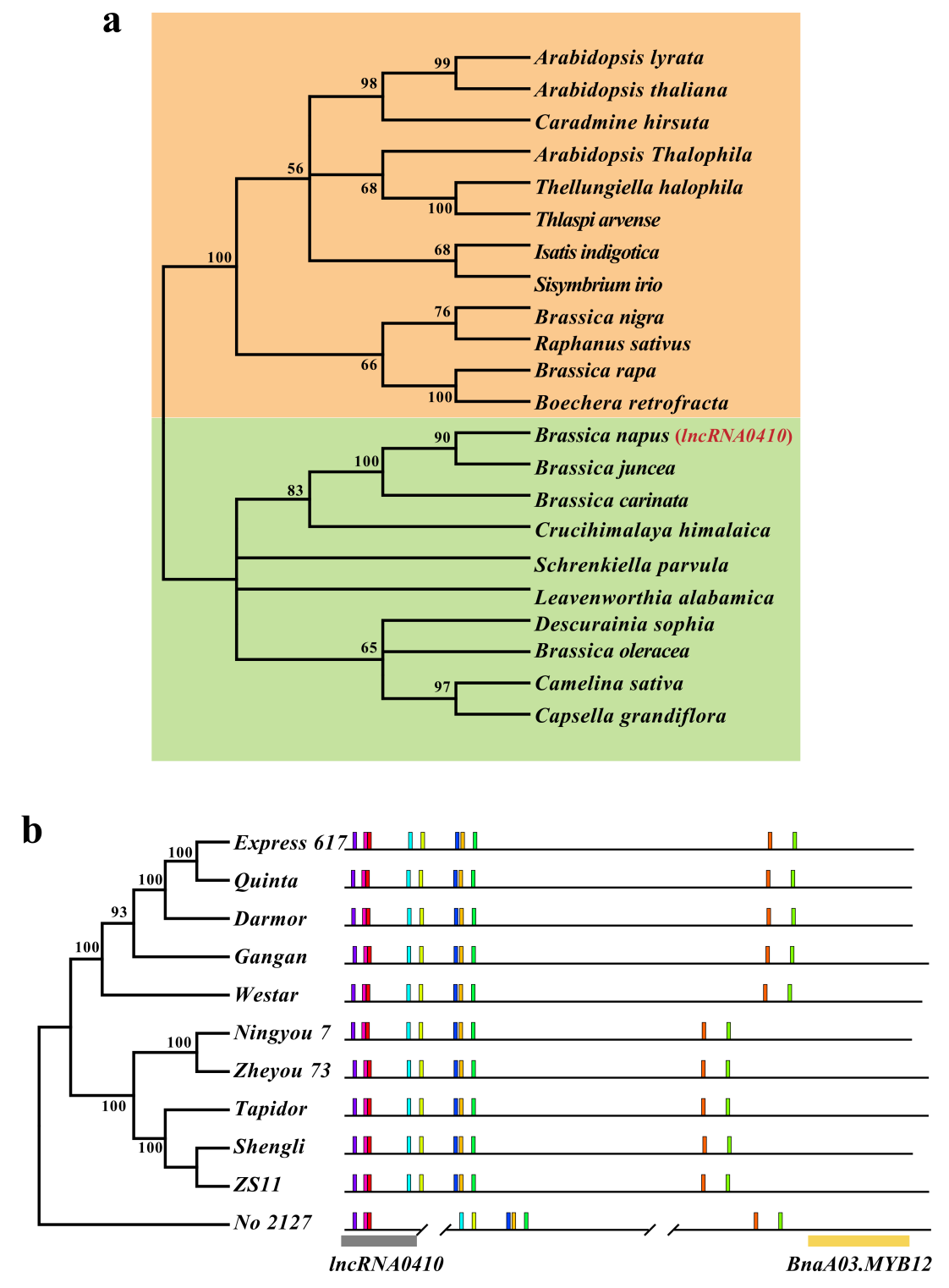


**Figure S11. Evolution of *lncRNA0410* in Brassicaceae species.**

**(a)** The phylogeny tree showed different Brassicaceae species used to identify *lncRNA0410*. A phylogenetic tree was constructed using the neighbor-joining method. The genome information for each species is at http://brassicadb.cn/#/. **(b)** Phylogenetic analysis of various *B. napus* genomes was conducted to identify *lncRNA0410* and its target gene *BnaA03.MYB12*. A neighbor-joining method was employed to construct the phylogenetic tree, with detailed genome information for each species provided at https://yanglab.hzau.edu.cn/.


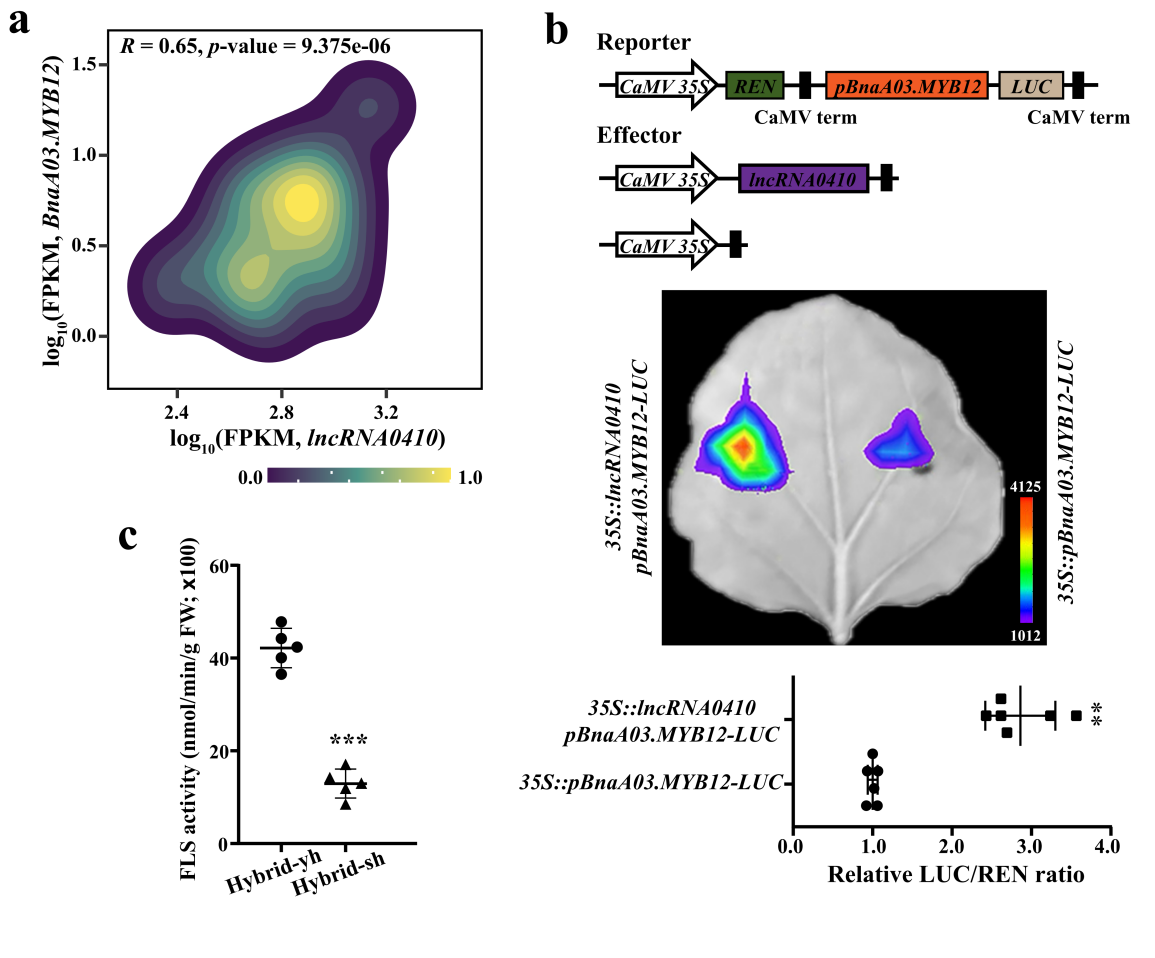


**Figure S12. *lncRNA0410* positively regulated the expression of *BnaA03.MYB12*.**

**(a)** The density plot represented the expression of *lncRNA0410* in F_1_ hybrids compared to *BnaA03.MYB12*. R is the Pearson's correlation coefficient. **(b)** The schematic diagram of the reporter and effector vectors for dual-luciferase assay. Dual-luciferase assays in *Arabidopsis* protoplast show that *lncRNA0410* transcriptional activates the expression of *BanA03.MYB12*. Data were means ±SD obtained from three biological experiments. Student’s *t*-test was used to calculate significance where ** indicated *p* < 0.01. Transient LUC imaging assays showing that *lncRNA0410* activates the expression of *pBanA03.MYB12-LUC*. **(c)** The graph showed flavonol synthase activity in Hybrid-sh and Hybrid-yh. Error bars indicated mean ±SD from five biological replicates. Student’s *t*-test; ***p* < 0.01.

**
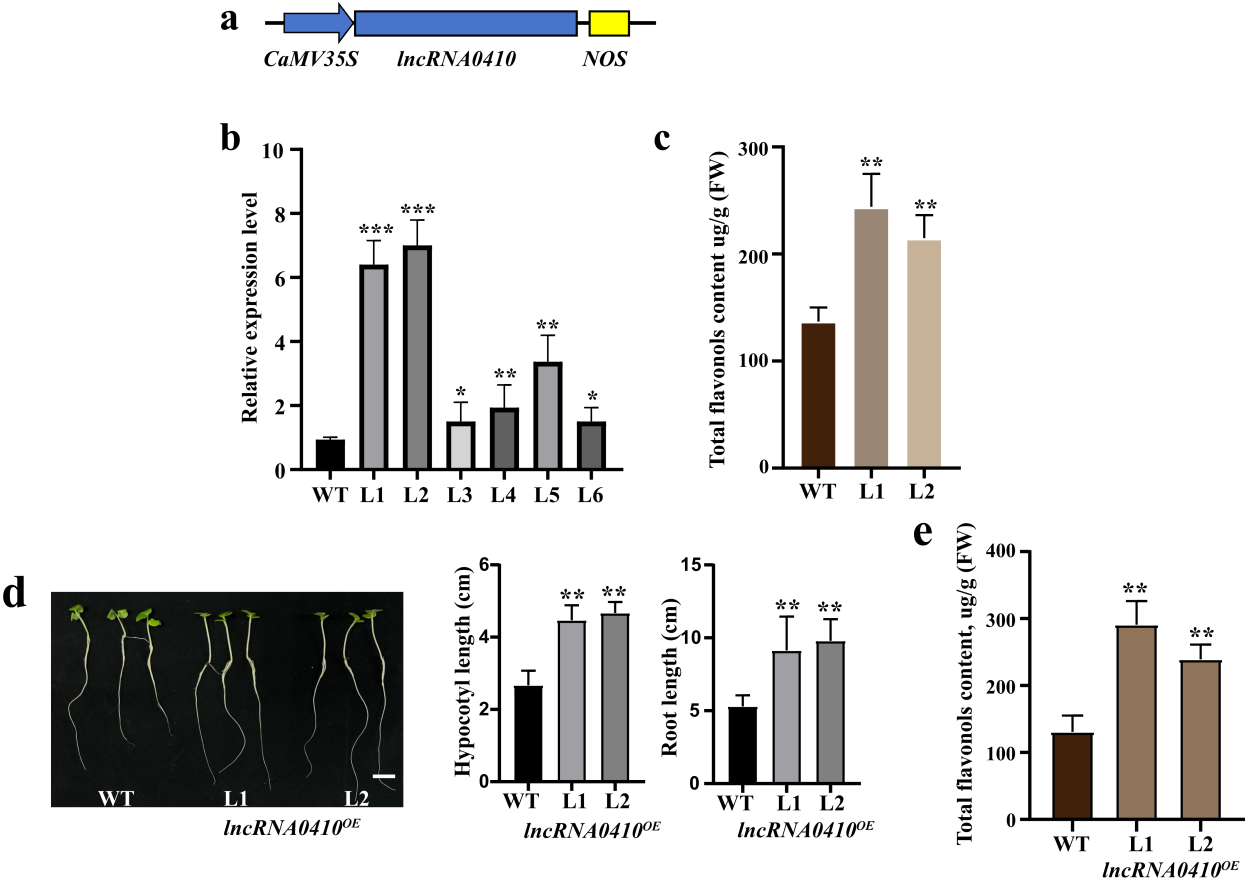
**

**Figure S13. Construction and phenotypic identification of *lncRNA0410*-overexpressing transgenic lines.**

**(a)** Schematic diagram of *lncRNA0410* (*XLOC_021109*) overexpression vector. **(b)** The graph illustrated the expression levels of *lncRNA0410* in wild-type (WT) and *lncRNA0410*^OE^ lines (T_0_ generation). The relative expression level of each gene was calculated using the 2^−ΔΔCT^ method (Livak and Schmittgen, 2001). The values were the mean ± SE of three biological replicates, with *BnaActin7* expression as an internal control. Student’s *t*-test was used to calculate significant differences (*, *p* < 0.05; **, *p* < 0.01; ***, *p* < 0.001). **(c)** The graph showed the total flavonols in WT and *lncRNA0410*^OE^ lines (T_0_ generation). The values were the mean ± SE of three biological replicates. Student’s *t*-test was used to calculate significant differences (**, *p* < 0.01). **(d)** Image (left) and graphs (right) showed hypocotyl and root lengths of WT and mutants (*lncRNA0410*^OE^-L1 and *lncRNA0410*^OE^-L2) at seedling stage. Error bars indicated mean ± SD from three biological replicates. Student’s *t*-test; ***p* < 0.01. **(e)** The graph showed the total flavonols in WT and *lncRNA0410*^OE^ lines (T_1_ generation). Error bars indicated mean ± SD from three biological replicates. Student’s *t*-test; ***p* < 0.01.


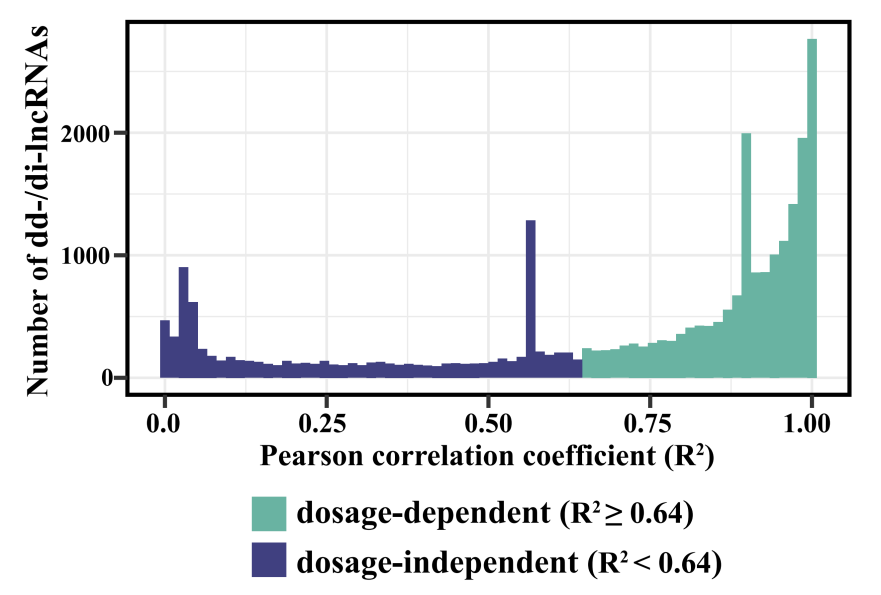


**Figure S14. Identification of dosage-dependent and dosage-independent lncRNAs.**

LncRNA with R^2^ ≥ 0.64 and R^2^ < 0.64 were deemed dosage-dependent and dosage-independent lncRNA.


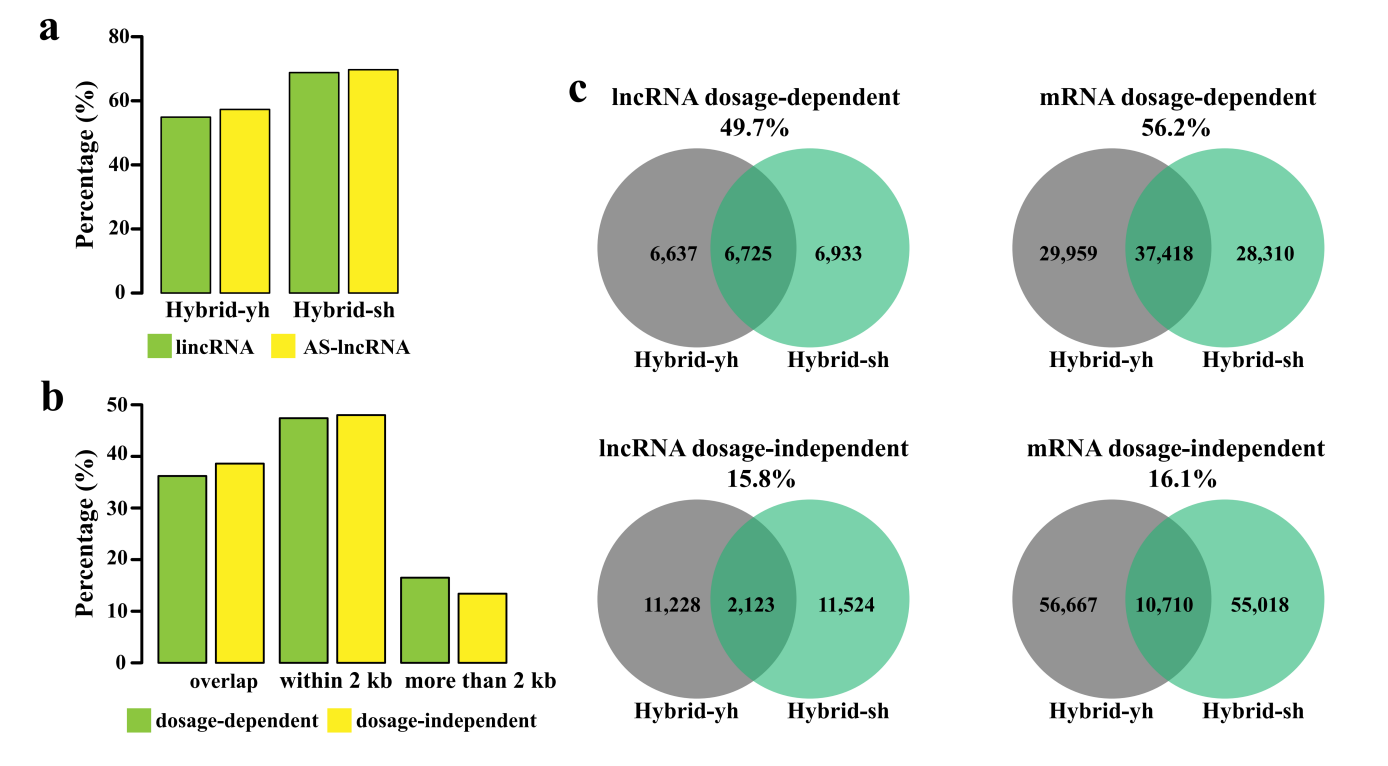


**Figure S15. Genome-wide dosage regulation of lncRNAs expression in F_1_ hybrids.**

**(a)** The graph showed the percentage of dosage-dependent AS-lncRNA and lincRNA in Hybrid-sh and Hybrid-yh. **(b)** Overlap with TE, within 2 kb of TE, and more than 2 kb with TE, dosage-dependent and dosage-independent ratios in lncRNA. **(c)** The percentage of lncRNA dosage-dependent, mRNA dosage- dependent, lncRNA dosage-independent, and mRNA dosage-independent overlap in two hybrids.
